# Association between single moderate to severe traumatic brain injury and long-term tauopathy in humans and preclinical animal models: a systematic narrative review of the literature

**DOI:** 10.1186/s40478-022-01311-0

**Published:** 2022-01-31

**Authors:** Ariel Walker, Ben Chapin, Jose Abisambra, Steven T. DeKosky

**Affiliations:** 1 Center for Translational Research in Neurodegenerative Disease, University of Florida, Gainesville, FL 32610 USA; 2 Department of Neuroscience, University of Florida, Gainesville, FL 32610 USA; 3 McKnight Brain Institute, University of Florida, Gainesville, FL 32610 USA; 4 Brain Injury, Rehabilitation, and Neuroresilience (BRAIN) Center, University of Florida, Gainesville, FL 32610 USA; 5 Department of Neurology, University of Florida, Gainesville, FL 32610 USA

**Keywords:** Traumatic brain injury, Tau, Moderate TBI, Severe TBI, Head injury

## Abstract

**Background:**

The initiation, anatomic pattern, and extent of tau spread in traumatic brain injury (TBI), and the mechanism by which TBI leads to long-term tau pathology, remain controversial. Some studies suggest that moderate to severe TBI is sufficient to promote tau pathology; however, others suggest that it is simply a consequence of aging. We therefore conducted a systematic narrative review of the literature addressing whether a single moderate to severe head injury leads to long-term development of tauopathy in both humans and animal models.

**Methods:**

Studies considered for inclusion in this review assessed a single moderate to severe TBI, assessed tau pathology at long-term timepoints post-injury, comprised experimental or observational studies, and were peer-reviewed and published in English. Databases searched included: PUBMED, NCBI-PMC, EMBASE, Web of Science, Academic Search Premiere, and APA Psychnet. Search results were uploaded to Covidence®, duplicates were removed, and articles underwent an abstract and full-text screening process. Data were then extracted and articles assessed for risk of bias.

**Findings:**

Of 4,150 studies screened, 26 were eligible for inclusion, of which 17 were human studies, 8 were preclinical animal studies, and 1 included both human and preclinical animal studies. Most studies had low to moderate risk of bias. Most human and animal studies (n = 12 and 9, respectively) suggested that a single moderate to severe TBI resulted in greater development of long-term tauopathy compared to no history of head injury. This conclusion should be interpreted with caution, however, due to several limitations: small sample sizes; inconsistencies in controlling for confounding factors that may have affected tau pathology (e.g., family history of dementia or neurological illnesses, apolipoprotein E genotype, etc.), inclusion of mostly males, and variation in reporting injury parameters.

**Interpretation:**

Results indicate that a single moderate to severe TBI leads to greater chronic development of tauopathy compared to no history of head injury. This implies that tau pathology induced may not be transient, but can progressively develop over time in both humans and animal models. Targeting these tau changes for therapeutic intervention should be further explored to elucidate if disease progression can be reversed or mitigated.

**Supplementary Information:**

The online version contains supplementary material available at 10.1186/s40478-022-01311-0.

## Introduction

Traumatic brain injury (TBI) is defined as a physical impact to the head that disrupts the physiological function of the brain [16, 31]. Every year, around 69 million people worldwide experience a TBI, and in the United States, approximately 5.3 million individuals are living with a TBI-related disability [16, 65, 87]. This places a significant emotional and economic toll on society and a compelling need to understand the sequelae that occur after a brain injury, since no treatments or cures exist [64]. TBIs can be categorized as mild, moderate, or severe and can lead to a variety of negative outcomes, such as changes in consciousness, loss of memory, alterations in behavior and mood, and motor impairments [31]. Falls are the leading cause of TBIs, significantly affecting children up to 17 years of age and the elderly aged 65 years and older [74]. TBIs also occur from motor vehicle accidents, sports-related injuries, blasts from explosions in war, or from being struck by or against an object (deceleration injury).

Post-mortem brain samples from individuals with a history of head injury display multiple proteinopathies, which are also associated with age-related neurodegenerative diseases [80]. It remains controversial if the proteins that accumulate post-TBI are due to the injury itself or are an unrelated product of other processes associated with aging. One of the proteins that has been shown to accumulate post-injury is tau. Tau is a physiologically hydrophilic soluble protein that functions to promote the formation and stabilization of microtubules in neuronal axons [27]. Tau binding to microtubules is regulated by various post-translational modifications, including phosphorylation [27]. In tau-associated neurodegenerative diseases (tauopathies), such as Alzheimer’s Disease (AD), tau is abnormally hyperphosphorylated and aggregated into insoluble fibrillary structures, which have been shown to strongly correlate with cognitive decline [30, 33]. The spread of tau pathology in tauopathies, specifically AD, has been grouped into six stages: Braak Stage I/II, tau is found in the locus coeruleus and transentorhinal/entorhinal regions; Braak Stage III/IV, tau spreads into the hippocampus and medial temporal lobe; Braak Stage V/VI, tau infiltrates the neocortex [12, 13].

The development of tau pathology post-injury has been heavily studied in individuals who sustained repetitive mild TBIs (rmTBI). The initiation of rmTBI studies began in 1928, when Dr. Harrison Martland coined the term “Punch Drunk” syndrome based on his observations of boxers who sustained rmTBIs [43]. These boxers developed changes in affect, cognition, and movement, and on postmortem examination showed evidence of brain damage [43]. The terminology of “Punch Drunk” syndrome has evolved through a number of terminologies over the ensuing decades; currently, the most common term being used is Chronic Traumatic Encephalopathy (CTE). This term has been applied to National Football League players who sustained rmTBIs and developed dementia and tau pathology. This term has also been applied to others who sustained rmTBIs, such as war veterans, who suffered from both impact injuries and blast wave injuries, and were shown to develop dementia and tau pathology [46, 55].

Mild TBIs are the most common form of TBI severity and are associated with ~ twofold increased risk of dementia in later life [7, 20, 22, 45, 47, 73]. Moderate to severe TBI (msTBI), however, correlates with a 2-to-fourfold increased risk for developing subsequent dementia (beyond the effects of physical brain damage that occur) [67]. The underlying mechanism linking msTBI with increased risk for dementia remains unknown [28, 67]. Because the progression of tau pathology correlates strongly with degree of cognitive impairment and is linked to multiple neurodegenerative diseases, it is vital to understand the long-term consequences of a single msTBI, with a specific focus on alterations in tau. Several studies have investigated tau pathology after msTBI, but reported conflicting results. A recent human imaging study assessed tau pathology in individuals who sustained a single msTBI and found that tau aggregation was present decades after injury [26]. Another study found that a single msTBI does not result in chronic tau pathology, creating discord in the literature surrounding msTBI and its relationship to tau [6]. Our review question was “does msTBI exposure increase risk for developing long-term tau pathology?” Currently, there are no reviews that have addressed this question in humans or animal models; therefore, the objective of this systematic review was to determine the association between a single msTBI and the development of long-term tau pathology. In humans, the population of interest is individuals who have sustained a single msTBI compared to humans without a history of TBI. In animals, the studies of interest were those in which animals received an analogous single msTBI compared to control animals that received a sham injury (undergoing all procedures applied to injured mice except the direct brain injury). Finally, the outcome was the long-term development of tau pathology, which we defined as changes in tau that were not physiological (hyperphosphorylated tau, insoluble tau, truncated tau, misfolded tau). Thus, the study included experimental studies for animal models and observational studies for human studies.

Through this systematic review, we sought to determine if tau pathology persisted chronically after moderate to severe brain injury. Findings from this review will help identify individuals with an increased risk for developing dementia following a single moderate to severe injury. Further, it will help guide development of targeted therapies to mitigate the effects of tauopathy disease progression, as well as reduce short and intermediate adverse outcomes of the TBI.

This systematic review was prepared in accordance with the reporting guidelines provided in the Preferred Reporting Items for Systematic Reviews and Meta-Analysis (PRISMA) statement [58, 59].

## Methods

### Eligibility criteria

We included studies that contained a single msTBI in humans and animal models and assessed tau pathology [utilizing blood samples, cerebrospinal fluid (CSF) samples, brain imaging, biochemical and immunohistochemical assessments etc.] compared to control cohorts with no history of TBI. We assessed tau pathology at long-term time points post-injury [for human studies this was defined as 6 months (mo) or greater post-injury; for animal models this was defined as 3mo or greater post-injury]; had experimental study designs or observational study designs (case control studies, cross-sectional and longitudinal cohort studies); and were peer-reviewed and published in English. Eligibility criteria were dependent on neither the age at which the msTBI was sustained nor the publication date. For human studies, the 6mo or greater time point was defined as long-term post-injury because that is regarded as the typical timespan for resolution of symptoms post-injury. In some individuals, post-concussive syndrome (PCS) can develop, consisting of changes in physical, cognitive, behavioral, and emotional behavior that occur after TBI [62]. While PCS typically resolves within a month or so, they can persist in some individuals beyond 3mo post-injury [62]. In addition, tau pathology can develop progressively with age, and is commonly present in subjects over 65 years of age [15]. Therefore, after discussion with co-authors, a 6mo or greater time point post-injury was chosen. For animal studies, the 3mo or greater time point was defined as long-term due to the shortened lifespan of most animal models. Typical mouse and rat models live a maximum of 24-36mo before dying of natural causes. The 3mo or greater time point was also chosen due to the progressive nature of tau pathology development in animal models, both transgenic and non-transgenic [19, 39]. Unpublished studies were excluded because we sought to assess peer-reviewed data for strength and quality of evidence that non-published studies might lack. Conference abstracts were excluded from the review, but were screened to assess if a full publication was available. If a full publication was found that fit the eligibility criteria, it was included in the review. Review articles identified in the literature search were excluded, but their references were examined for any articles missed. Studies that could not be translated into English were excluded. We included case studies without control groups, but results from these studies were not included in the main interpretation if a single msTBI leads to chronic tau pathology.

### Information sources

Our comprehensive literature search was first conducted on November 3, 2020, from the following electronic sources: PUBMED; PMC database on NCBI; EMBASE; WOS, BCI, CABI, KJD, MEDLINE, RSCI, SCIELO, ZOOREC databases on Web of Science; and Academic Search Premiere. On November 8, 2020, APA PsychNet was searched.

### Search Strategy

The search strategy for each information source was based on keywords for traumatic brain injury (head injury, brain injury, or TBI), which were combined using “OR”, followed by the outcome of interest (tau pathology), which was joined using “AND” in the search strategy. Some searches also included the phrase “single moderate to severe” to specify the type of exposure. Using this phrase on some information sources resulted in exclusion of articles that should have been assessed for inclusion and therefore this phrase was deleted from that search strategy. There were no limits applied to the search strategy. If multiple databases from an electronic source were searched, the same search strategy was applied to all of them. The specific search strategies for each information source were:

- PUBMED: "single moderate to severe" AND "traumatic brain injury" OR "brain concussion" OR "brain contusion" OR "TBI" AND "tau"
- PMC-NCBI: "single moderate to severe" AND "traumatic brain injury" OR "brain concussion" OR "brain contusion" OR "TBI" AND "tau"
- EMBASE: traumatic AND ('brain'/exp OR brain) AND ('injury'/exp OR injury) AND single AND moderate AND to AND severe AND tau
- Web of Science: TS = (single moderate to severe AND traumatic brain injury OR TBI AND tau), Timespan = All years, Search Language = Auto
- Academic Search Premiere: (traumatic brain injury or head injury, or brain injury or tbi) AND tau
- APA PsychNet: Traumatic brain injury and tau (in All Fields)

### Selection process

All database search references were uploaded to and managed through Covidence® in the review process. Duplicate references were removed automatically by Covidence®. To select studies, all uploaded references went through two screening processes. The first screening process consisted of going through titles and abstracts; titles and abstracts that did not contain the inclusion criteria were excluded, while those that did went through the second screening process. This process consisted of reading the full text in order to select articles that contained eligibility criteria. The first screening process was done independently by one reviewer (AW) and the second screening process was performed by two independent reviewers (AW and BC). Articles about which there was disagreement between the two independent reviewers were reviewed by a third reviewer (either STDeK or JFA) for a final decision of either inclusion or exclusion based on the eligibility criteria.

### Data collection process

Data extraction from articles included in this review was performed independently by one reviewer (AW). Data from each article were copied into an excel file. No automation tools or software programs were used to collect or extract data and no articles included in this review needed translation.

### Data items

For human-based studies, data extraction consisted of the study design, exposure severity (moderate and/or severe injury), injury rating, type of injury, sample sizes for both the TBI cohort and the comparators (individuals with no history of TBI), the number of males and females in each group, ages for the TBI and comparator cohorts at the time of the study, inclusion and exclusion criteria for samples or participants in the study, time since the injury, and outcome (type of tau assessment). For the animal studies, data extraction consisted of the type of animal model (species and strain), injury severity, injury rating, injury model (fluid percussion injury (FPI), controlled cortical impact (CCI), ect.), injury parameters, sample sizes for both TBI and sham groups, age at injury, survival time (or time since injury), and the outcome (type of tau pathology). We reported for both human and preclinical animal studies how tau was measured/collected, such as through biospecimen (blood, CSF, or saliva), imaging (Positron emission tomography (PET) and type of PET ligand), or through post-mortem examination (ELISA, SiMoA, staining using various antibodies, or western blots). In addition, how the studies classified the TBI severity was collected, such as with the Glasgow Coma Scale or through self-report for human studies or through behavioral tests for animal models.

### Risk of bias in individual studies

Assessing risk of bias in individual studies was done both at the study and outcome levels. For human studies, excluding the case studies, the Newcastle–Ottawa Scale (NOS) was used to assess the risk of bias in cohort studies and cross-sectional studies [56]. The NOS assesses the risk of bias in individual studies by evaluating three categories: selection, comparability, and outcome. Each category can receive a maximum of 2–4 stars. The more stars a category receives, the smaller the risk of bias in that category, while fewer stars equate to a higher risk of bias. Within the selection category, there are four sub-assessments, resulting in a maximum of four stars for the selection category. Sub-assessment number four, which evaluates if the study demonstrates that the outcome of interest was not present at the start of the study, was deemed non-applicable for assessing risk of bias in our included studies. Our outcome of interest was the long-term development of tau pathology and since most human studies assessed tau pathology post-mortem or retrospectively, this was not possible to address; therefore, for the selection category, a maximum of three stars could be allotted for articles included in this review. For the comparability category, the NOS allows authors to select the most important factor and any additional factor(s) to be considered for assessing the comparability of cohorts. We defined the most important factor for comparability of cohorts to be history of head injury. The second additional factor for comparability of cohorts was family history of dementia or other neurological diseases that could affect tau pathology. If individual studies controlled for both factors, two stars were given. The last category, outcome, has three sub-assessments for a maximum of three stars. For our specific objective of this review, we deemed sub-assessment number three as non-applicable. This assessment evaluated the adequacy of follow-up of cohorts, but for studies included in this review, there were no follow-up time periods; therefore, for the outcome category a maximum of two stars could be allotted. Risk of bias was done independently by two reviewers (AW and BC) for all articles included in this review. Any disagreements on the assessment of the risk of bias were given to a third reviewer (STDeK or JFA) for final evaluation.

Well-established scales or checklists for assessing the risk of bias in preclinical studies are not available; therefore, the National Institute of Health principles and guidelines for reporting preclinical research on rigor and reproducibility was used to guide interpretation/risk of bias and strength of results obtained in preclinical animal studies. These guidelines outline several key principles, such as rigorous statistical analysis, transparency in reporting, and establishment of best practice guidelines for specific methods.

### Data synthesis

For potential quantitative assessment of studies, continuous data when reported were extracted. Due to heterogeneity in study and outcome levels, a meta-analysis was deemed not appropriate and could not be conducted. Therefore, a qualitative and descriptive/narrative summary of the findings are reported in this systematic review.

## Results

### Study selection

**Fig. 1:**
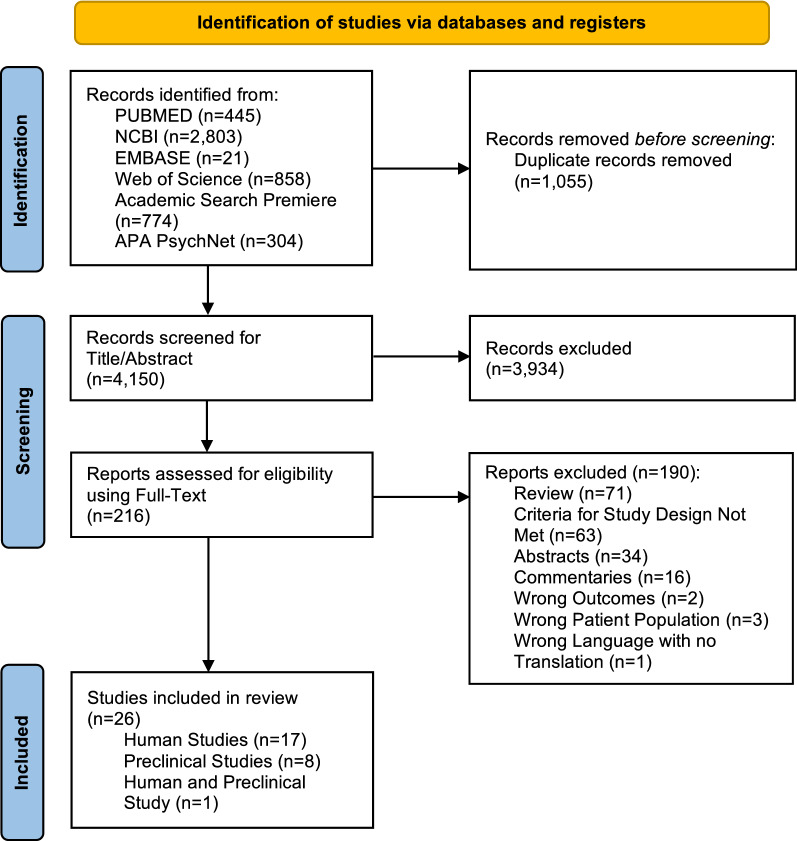
PRISMA 2020 flow diagram for study selection process and outcome

After searching all information sources, we found a total of 5205 articles through our specific search strategies. Once duplicates were removed, there were 4150 articles to screen for titles and abstracts. After the first screening, 216 articles met criteria for full-text screening. Once full-text screening was completed, 26 articles were eligible for inclusion in the review, of which 17 were human studies, 8 were preclinical animal studies, and 1 study included both humans and a preclinical animal model. A flow diagram is shown in Fig. 1 including reasons for article exclusion.

### Study characteristics

In this review, we assessed in both human and preclinical animal studies whether a single msTBI could lead to the long-term development of tau pathology. The individual human studies assessed in this review had the following study characteristics: type of study design, injury severity, how the injury was rated, injury type, sample size (for our specific population of interest) and number of males and females, age at time of assessment, inclusion and exclusion criteria for samples or the sample population recruited, post-TBI interval (time since injury), type of tau assessment, the findings of the individual studies, and if those findings supported the conclusion of long-term development of tau pathology. Additional files display these human study characteristics for each article in table format (see Additional Files 1, 2, 3, and 4). For quantitative studies, continuous data reported were included in the “findings” column. The study characteristics from the individual preclinical animal studies, which reflected most of those from the human studies, additionally included the type of animal model used in the study and the specific injury parameters for the injury model used. Additional files display these preclinical animal study characteristics in table format (see Additional Files 5, 6).

### Risk of bias in studies

**Fig. 2:**
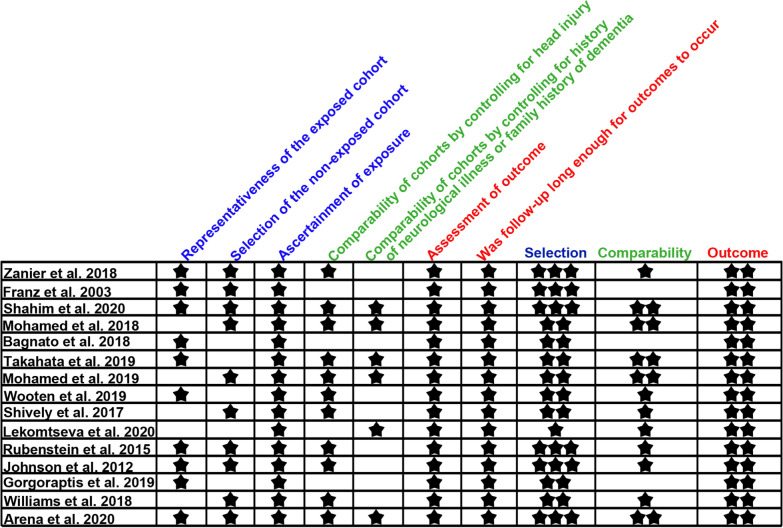
Risk of bias assessment in human studies using NewCastle–Ottawa Scale

Risk of bias assessment for human observational studies was assessed using the NOS [56]. A summary of these assessments is provided in Fig. 2. With the NOS, a judgement of overall risk of bias assessment per article is not integrated into the scaling system. Instead, a “star system” is used in which a study is judged on three broad perspectives: selection of study groups (blue), comparability of the groups (green), and ascertainment of the outcome of interest (red). The more stars a category receives the smaller the risk of bias, whereas fewer stars equate to a higher risk of bias. For selection, a maximum of three stars can be given, for comparability a maximum of two stars, and for outcome a maximum of two stars can be given. Since all studies included in this review received the maximum two stars for the outcome category, a text summary is provided below for only the selection and comparability category components of the risk of bias assessment.

For the selection category, 6 of the 15 studies (6/15) received all three stars, meaning low risk of bias [4, 23, 34, 61, 66, 88]. Eight of the 15 studies received two stars for the selection category either because the exposed cohort was not representative of the general population (the exposure or TBI was not a typical TBI occurring in the general population), or the selection of the non-exposed cohort was not drawn from the same community as the exposed cohort [6, 26, 49, 50, 68, 76, 83, 84]. Typically, TBIs representative of the general population include falls, motor vehicle accidents, or assaults [41]. In three of these studies (3/8), the TBI resulted from war-related injuries [49, 50, 83], while in one study (1/8) the TBI was modeled by a prefrontal leucotomy [68]. In the other four studies (4/8) that received two stars for the selection category, the non-exposed cohort was not selected from the same community as the exposed cohort. One of these studies did not have a sampled control group, but instead compared tau outcomes with normalized values taken from a separate cohort from a different study [6, 71, 81]. Two of these studies recruited the control group from a different community than the exposed cohort [26, 76], and the other did not state the source of the controls [84]. Only 1 out of the 15 studies included in this review received one star for the selection category, meaning high risk of bias [40]. The authors did not disclose of the cause of the TBI in the exposed cohort, making determination of the representativeness of the exposure impossible, and the recruitment source of the patients and controls was not specified [40].

In the comparability category, 5 of the 15 studies received the maximum score (2 stars), meaning low risk of bias [4, 49, 50, 66, 76]. A majority of the studies (7/15) received 1 star in the comparability category, due either to not controlling for the primary factor for comparability between TBI and controls (head injury) [40], or for not controlling for the second factor (history of neurological disease or family history of dementia) [34, 61, 68, 83, 84, 88]. Three of the 15 studies received no stars for the comparability category, indicating a high risk of bias. One of these studies did not state that the control groups lacked a history of TBI and those control groups had either headaches or dementia [23]. The second study did not state that the control grouped lacked a history of head injury and the authors did not define “healthy” for the control group, making it unclear if the control group lacked a history of neurological illnesses or a family history of dementia [26]. The other study had no control group, but used normalized tau values from another study using healthy individuals or individuals with AD or dementia with Lewy bodies [6, 71, 81]. The demographics of these healthy individuals or demented patients did not state, though, if there was no history of TBI [71, 81].

Since checklists or scales to assess risk of bias in preclinical studies have not been fully developed yet, this review utilized the NIH’s principles and guidelines for reporting preclinical research. This outlines core principles that preclinical studies should disclose of to enhance rigor, reproducibility, and transparency, including rigorous statistical analysis, transparency in reporting statistics, randomization, blinding, sample size estimation, inclusion and exclusion criteria, and appropriately disclosing of all biological material.

For statistical reporting, all 9 preclinical animal studies disclosed of the statistical test used, the value of N, and description of the study’s center, dispersion, and precision measures. For transparency in reporting randomization, most studies reported that animals were randomly assigned to either the experimental or sham treatment [2, 21, 24, 37, 38, 69, 88], while 2 out of the 9 studies did not state randomization [1, 77]. For those studies that did report randomization, the method used to randomize animals into treatment versus control groups was not reported. All preclinical animal studies had experimenters blinded to group assignment and outcome assessment.

The NIH’s principles and guidelines for reporting preclinical research states that authors should mention whether an appropriate sample size was computed during the study design and which statistical method was used to compute this. If no power analysis was used, the authors should state how the sample size was determined. None of the preclinical animal studies included in this review calculated sample size using power analysis, but some studies (3 out of 9) estimated the sample size based on the variation and mean of the samples or from a previous study [2, 38, 88]. Six out of the 9 preclinical studies did not compute or state how the sample size was determined, leading to most studies having a small sample size [1, 21, 24, 37, 69, 77].

For transparency in inclusion and exclusion criteria, most of the preclinical studies (7/9) did not justify only using male animals and excluding female animals [1, 2, 24, 38, 69, 77, 88], with only one study using both sexes and another study not mentioning the sex of the animals used [21, 37].

Lastly, the NIH principles and guidelines for reporting preclinical research state that the description of biological materials should disclose of enough information to identify the reagents. For antibodies, studies should report the source, characterization, dilution, and how they were validated. Most of the preclinical animal studies included in this review reported the source from which the antibodies were bought, but there were inconsistencies in reporting the dilutions of antibodies, the characterization/specificity of the antibodies, and no studies reported how the antibodies were validated. This raises concerns for reproducibility. For animals, the NIH guidelines state that the source, species, strain, sex, age, husbandry, and inbred and strain characteristics of the animals should be reported. All studies reported the source, strain, and species used in the study, but there were inconsistencies in reporting the sex, age, husbandry, and inbred and strain characteristics.

### Results of individual studies

There were a total of 18 reports that included human assessment of tau pathology chronically post msTBI, while only 9 articles assessed this in preclinical animal models. Of the human studies, 13 articles were observational cohort studies, 2 were observational cross-sectional studies, and 3 were case studies. All preclinical animal studies had experimental study designs.

Due to high variability and heterogeneity in reporting effect measures, a meta-analysis could not be conducted; therefore, a systematic narrative summary of the results is presented. Human studies were grouped by “study design” containing the highest level of quality of evidence presented first, observational cohort studies (n = 13), followed by observational cross-sectional studies (n = 2) and then case studies (n = 3) [75]. Preclinical studies were grouped by type of animal model, including C57BL/6 mice (n = 4), rats (n = 3), and transgenic mouse models (n = 2).

### Human studies

#### Observational cohort studies

In Zanier et al., the objective was to assess if CTE pathology, mostly associated with repetitive mild concussions and blasts, could be a product of a single moderate to severe closed TBI. Upon neuropathological examination, PHF1(pS396/404) immunoreactivity in TBI patients surviving a year or more was observed in 12 out of the 15 cases [88]. Pathology appeared as neurofibrillary tangles (NFTs) and neurites, within astrocytes, and plaques of all cortical layers, but more prominent in superficial layers of the cortex [88]. All sectors of the hippocampus demonstrated PHF-1 immunopositivity. PHF1 immunopositivity also appeared in 12 out of the 15 uninjured control cases, but the extent and distribution of tau pathology was more widespread in the TBI group compared to the controls [88]. Specifically, PHF-1 tau was restricted to the entorhinal cortex (ERC) in most of the controls with only four of them displaying extensive pathologies extending to the hippocampus and cortex [88]. These four controls, though, were older in age (79 years +). PHF-1 tau observed in the TBI group was dispersed in moderate to high density throughout the medial temporal lobe, including the hippocampus, and extending out to the cortex, and this was observed at younger ages compared to controls (59 years +) [88]. Further, three of the 12 TBI cases displaying PHF-1 immunoreactivity displayed CTE-like patterns with pTau found in both neurons and glia cells clustering around cortical vessels, with two of the three cases displaying this at the depths of sulci [88]. Altogether, these results suggest that a single msTBI may not only lead to more widespread phosphorylated tau (pTau) deposition, but also an acceleration of tau pathology.

Franz et al., assessed if amyloid beta 1–42 and tau in the CSF could have predictive value for prognosis after TBI. For this review, only data on tau in the CSF was extracted. The authors discovered that total tau levels in the CSF collected from 43 to 284 days post-trauma were in the normal total tau level range compared to controls with headache and dementia [23]. This study’s result suggests that total tau levels at chronic time points post-severe TBI are not affected.

In Shahim et al., the objective was to determine whether specific proteins measured in the serum of patients with TBI, including tau, were related to TBI diagnosis, injury severity, brain volume, and diffusion tensor imaging (DTI) measures of traumatic axonal injury. Longitudinal assessment of total tau levels were increased significantly in severe TBI patients compared to controls at the 1 and 2 year time points, but not at the 3, 4, or 5 year time points [66]. These results suggest that total tau in the serum is affected in a time-dependent manner, with tau increasing significantly after TBI, but then returning to normal levels at more chronic time points.

Mohamed et al. attempted to determine if TBI and post-traumatic stress disorder (PTSD) in individuals who served in the Vietnam War increased amyloid beta accumulation in the brain using amyloid PET and how the degree of change would correlate with tau and amyloid beta concentrations in the CSF. For this review, we extracted data only on CSF total tau and pTau (Thr181). The study assessed four cortical regions of interest: frontal, temporal, cingulate, and parietal. Of these four regions, only two regions, the frontal and temporal cortex, showed a significant positive correlation between amyloid beta in the brain and CSF tau-protein concentrations in the healthy controls [50]. The msTBI group, though, showed significant positive correlations between amyloid beta in the brain and CSF tau-protein concentrations in all four regions of interest, implying that a single msTBI may lead to abnormal changes of tau in the CSF decades after brain injury [50].

In Bagnato et al., the objective was to investigate changes in CSF amyloid beta 1–42, total tau, and pTau (Thr181) in the chronic phase post-TBI of individuals who developed prolonged disorder of consciousness after a severe TBI. For the purposes of this review, only data on total tau and pTau were extracted. Instead of using control samples, authors used reference normal values of total tau (< 300 pg/ml) and pTau (< 61 pg/ml) from a previous study that were obtained from healthy individuals aged 21–93 years for comparison [71]. Authors found that in all 15 patients, normal total tau levels were observed in the chronic phase post-TBI and 14/15 patients had normal pTau levels [6]. Only one patient had slightly higher levels of pTau in the CSF compared to normal reference values [6]. These results suggest that in the chronic phase post severe TBI, individuals who develop prolonged disorder of consciousness have normal total tau and pTau (Thr181) levels.

In Takahata et al., the objective was to assess the association between the topology of tau lesions with late-onset psychiatric symptoms in long-term (> 2 years) survivors of repetitive mild and severe TBI compared to healthy controls with no history of TBI or other neurological or psychiatric diseases. PET imaging using ^11^C-PBB3, which binds to NFTs, revealed no significant differences between rmTBI and severe TBI in the white and gray matter; therefore, rmTBI and severe TBI data were combined when compared to healthy controls [76]. In the neocortical gray matter, TBI patients had significantly higher ^11^C-PBB3 tau binding compared to healthy controls, but not in the subcortical gray matter [76]. Regional assessment within the gray matter revealed that TBI patients had significantly higher ^11^C-PBB3 tau binding in the temporal and occipital gray matter segments compared to healthy controls [76]. Within the entire white matter segment, TBI patients had significantly higher ^11^C-PBB3 tau binding compared to healthy controls [76]. Further, when assessing regions within the white matter, the TBI group had significantly more tau binding in the temporal, frontal, and occipital lobes compared to healthy controls [76]. The authors then assessed sub-regional differences in ^11^C-PBB3 tau binding in the frontal white matter and found that TBI patients had significantly more binding in the medial frontal white matter only [76]. It was also found that TBI patients had significantly more tau binding in the surface of the white matter, but not in the deep white matter subsegment, compared to healthy controls [76]. These results suggest that TBI leads to region specific vulnerabilities to ^11^C-PBB3 tau pathology in the chronic phase after severe injury.

In Mohamed et al., the primary objective was to evaluate tau accumulation, using the PET ligand [^18^F]AV1451 (flortaucipir), in the brains of cognitively normal male Vietnam war veterans who had a history of moderate/severe non-penetrating TBI and/or PTSD compared to Vietnam war veterans with no history of TBI and/or PTSD. For the purposes of this review, only data from TBI (without PTSD) and the normal controls were extracted. The TBI group had significantly higher [^18^F]AV1451 tau uptake in the superior frontal and middle frontal gyri, medial and lateral orbitofrontal cortex, precentral and postcentral gyri, insula, supramarginal gyrus, precuneus, superior temporal gyrus, transverse temporal gyrus (Heschel’s gyrus), temporal pole, and basal ganglia compared to control. The left inferior temporal gyrus had significantly lower [^18^F]AV1451 tau uptake in the TBI vs. control group [49]. When assessing [^18^F]AV1451 tau uptake in the frontal, parietal, and temporal lobes and cingulate cortex, the TBI group only showed significantly higher uptake in the frontal cortex compared to controls. The authors estimated the Braak stages of the TBI cases compared to controls based on the tau PET imaging data and the PET amyloid beta positivity. In the TBI group, three were amyloid negative and were either in Braak stage 0 or I/II (n = 1 for stage 0, n = 1 for stage I, and n = 1 for stage II) compared to the healthy controls that were amyloid negative (n = 13) of which 10 were in Braak stage 0 and 3 were in Braak stage II [49]. In the 7 amyloid positive TBI subjects, 2 were in Braak stage I, 2 were in stage II, 2 were in stage III, and 1 was in stage IV compared to amyloid positive healthy controls (n = 8), of which 4 were in Braak stage 0 and 3 in stage II with only 1 individual in stage IV [49]. These results suggest that a non-penetrating msTBI can lead to the long-term development of regionally specific tau deposition in an AD-typical profile (Braak stages I-IV) in addition to atypical-AD regions, such as the frontal and cingulate cortex. Since the mean age for TBI cases was 72.6 years and for controls 74.29 years, the AD-like pattern of tau deposition observed might be indicative of AD on the rise.

Wooten et al. utilized ^18^F-Flortaucipir PET, which images paired helical filaments of pTau, in conjunction with magnetic resonance imaging (MRI) and DTI to assess interrelationships among tau deposition, white matter integrity, and gray matter functional connectivity in a population of TBI subjects. For this review, we extracted data only from the ^18^F-Flortaucipir PET imaging of the 34-year-old male subject who sustained a single severe TBI due to an automobile accident 2.5 years prior to the study. This subject demonstrated the highest binding of ^18^F-Flortaucipir distribution volume ratio (DVR), compared to all other subjects, in the corpus callosum extending into the posterior cingulate and in the thalamus and brainstem [84]. These results suggest that a single severe TBI can lead to regional increases of tau in the brain, but since only one subject in this study sustained a severe TBI, interpretation of these results should be done cautiously.

In a study of tau accumulation in schizophrenia, five patients who had undergone bilateral prefrontal leucotomy (a model of single severe axonal injury) were compared to five non-leucotomized schizophrenia patients [68]. In all leucotomy cases, there were qualitative increases in pTau (AT8) and NFTs in the cortex at the leucotomy site compared to only 1 out of 5 non-leucotomy cases in control tissue [68]. The prefrontal cortex rostral to the leucotomy site of 2 out of the 5 leucotomy cases displayed scant pTau (AT8, CP13, and PHF-1) immunoreactivity and NFTs compared to only 1 out of 5 non-leucotomy cases [68]. Only 1 out of the 5 leucotomy cases displayed scant NFTs and pTau (AT8 and CP13) in the frontal cortex caudal to the leucotomy site; none of the control cases displayed this pathology [68]. In most of the leucotomy cases (4 out of 5), the pattern of tau deposition in the prefrontal cortex around the leucotomy site resembled that observed in CTE, with AT8 and CP13 positive tau astrocytic tangles and neurites found at sulcal depths and around blood vessels [68]. Only one out of the five leucotomy cases displayed CTE-like pathology in the prefrontal cortex rostral to the leucotomy site [68]. Within the hippocampus, 3 of the 5 leucotomy cases displayed moderate NFTs in the CA1 region and some in the subiculum and ERC, whereas the other 2 leucotomy cases had only rare isolated NFTs in the CA1 region. Thus, in these particular cases of severe TBI (predominantly an axonal injury), a majority developed CTE-like pTau pathology near the surgical site, and to an extent, also in brain areas distal to the injury site.

Lekomtseva et al., sought to identify protein levels in the blood associated with mitochondrial dysfunction, including tau, one year after moderate TBI. At this point, total serum tau levels were higher but not significantly different than controls [40].

Rubenstein et al. utilized a novel immunoassay using multi-arrayed fiberoptics (EIMAF) to detect total tau and pTau in the serum of individuals who sustained a severe, blunt TBI. This proof-of-concept study demonstrated that this assay identified proteins of interest with the most sensitive detection limit (attogram per milliliter (ag/mL)) and that this assay could be applied to determine tau biomarkers for diagnostic purposes [61]. Six months after severe TBI, total tau levels in serum were not statistically different from control samples, but pTau remained significantly higher than in controls [61]. While a proof-of-concept study, these results imply that tau dynamics may be differentially affected in the chronic phase post-TBI, with pTau remaining elevated for a longer time period, compared to total tau.

We extracted tau pathology data from a study of long-term tau and amyloid beta pathology present after a single msTBI compared to non-injured age-matched controls. NFT pathology in long-term (average ~ 8.2 years) survivors of TBI was observed across the entire age spectrum and was more common (~ 47%; 18 of 39 cases) than in controls, in whom NFT pathology was observed mainly in individuals over 60 years of age and was less common (~ 34%; 16 out of 47 cases) [34]. When comparing TBI to control cases that were less than 60 years old, NFTs were observed significantly more often in TBI (~ 34%; 11 of 32 cases; age range 19–60 years), compared to control cases (~ 9%; 3 of 32; age range 14–60 years). These three control cases were either 55 or 60 years old [34]. Further, long-term survivors of the single msTBI manifested tau pathology in a CTE-like pattern, with tau pathology at the depths of cortical sulci and in the superficial cortical layers [34]. Some TBI cases also demonstrated more extensive and widespread tau pathology (4 of 18) throughout the hippocampus, subiculum, and isocortical (cingulate/insular) regions compared to control cases that displayed NFT pathology restricted primarily to the transentorhinal cortex and CA1 region of the hippocampus (13 of 16) [34]. These results suggest that not only does a single msTBI accelerate tau pathology, but it may also induce widespread tau pathology in a CTE-like pattern.

Using flortaucipir PET imaging, Gorgoraptis et al. assessed whether tau pathology developed long-term after msTBI (n = 21) compared to healthy controls (n = 11). This in vivo imaging data was also correlated with neurodegenerative markers in the CSF, structural MRI, and cognitive performance. For this review, only tau data on imaging and CSF analysis were extracted. Several (15 of 21) TBI participants had more flortaucipir binding compared to healthy controls in the cortical and subcortical gray and white matter, while others (6 of 21) had low to absent levels of binding like healthy controls [26]. Overall, the TBI group had significantly more flortaucipir binding in the right lateral occipital cortex compared to the controls, but there were no significant differences in tau binding in the entorhinal, perirhinal, parahippocampal cortices or hippocampus between the TBI groups and controls [26]. CSF tau did not correlate with tau binding in the cerebral gray or white matter in the controls, but both CSF total tau and pTau (Thr181) correlated with tau binding in the cortical gray matter and white matter in TBI cases [26]. No correlations were found between total or pTau and flortaucipir binding in either group and no significant differences in total tau or pTau concentrations in the plasma or CSF between TBI and healthy controls [26]. These results suggest that tau pathology in the brain is influenced long-term post msTBI in a spatially dependent manner and that tau dynamics in the CSF and plasma may not distinguish between TBI and healthy controls.

#### Observational cross-sectional studies

In Williams et al., the primary objective was to assess markers of chronic injury in the sera of individuals who had sustained a war-related msTBI using 11 different panels of single chain variable protein fragments (svFvs), and to determine if the presence of these proteins indicated an increased risk of neurodegenerative disease. For this review, only outcomes on svFvs that bound to oligomeric tau (F9T and D11C) were extracted [78]. In these chronic post-TBI cases the svFvs F9T and D11C had significantly stronger reactivity in the sera of individuals with a single msTBI compared to sera of controls [83]. This significant increase in oligomeric tau in the sera long-term post single msTBI might be a potential indicator of high risk for developing neurodegenerative diseases.

Arena et al. assessed the various post-translational modifications and tau isoform compositions found in CTE neuropathologies from three long-term survivors of single msTBI. These changes in tau were compared to other tauopathies with no history of TBI exposure, such as AD, primary age-related tauopathy (PART), aging-related tau astrogliopathy (ARTAG), and various subtypes of frontotemporal lobar degeneration with tau inclusions (FTLD-tau). In the long-term survivors of a single msTBI, post-mortem assessment of tau revealed characteristic CTE pathology, with tau in both astrocytes and neurons in a patchy distribution at the sulcal depths and around blood vessels. This pattern was not observed in the non-injured cases of other tauopathies. Astrocytes in TBI cases were moderately immunoreactive for RD4 (4R tau) in two of the three cases (Cases 12 and 13), while 3R tau was absent from astrocytes in all three TBI cases, like what has been observed in ARTAG, progressive supranuclear palsy (PSP) and corticobasal degeneration (CBD) control cases [4]. NFTs were moderately to intensively immunoreactive for both 3R and 4R tau in two of the three TBI cases (Cases 12 and 13), similar to NFTs observed in non-injured control AD and PART cases [4]. PTau staining with PHF-1, AT100 (pT212/S214), and CP13 (pS202) was moderate to intense in both astrocytes and neurons in all three TBI cases. All 3 TBI cases had substantial pTau reactivity (pS262) only in neurons, although one case (Case 13) displayed moderate pS262 immunoreactivity in astrocytes [4]. These phosphorylated-tau pathologies aligned with observations in ARTAG and AD uninjured control cases. Minimal tau-C3, which assesses tau truncated at D421, was found in neurons of all 3 TBI cases, indistinguishable from the non-injured control ARTAG cases [4]. Finally, tau staining with conformational antibodies (GT-7 and GT-38) was moderately to strongly immunoreactive only in neurons of all three TBI cases, like that observed in the AD and PART cases [4]. These findings suggest that tau pathology is maintained in the long-term post TBI period, but only the pattern and distribution of tau pathology distinguishes TBI-related tau from other age-related and primary tauopathies.

#### Case studies

Okamura et al. determined changes in pTau levels in the brains of 2 schizophrenic patients who had undergone prefrontal leucotomy, a model of severe TBI [68]. Case 1 was leucotomized during adolescence, while Case 2 was leucotomized through the orbito-ventromedial cortex in her 20s [54]. Both survived over 53 years post-surgery. Neuropathological examination revealed considerable pTau immunostaining in neurons and glia in the frontal cortex as well as lesser staining in the cingulate gyrus, medial nucleus of the thalamus, and nucleus accumbens [54]. Neurofibrillary tangles and astrocytic tangles, unlike pTau, were less frequent in the cortex [54]. Perivascular pTau aggregates (pS422) were seen occasionally, but prominent astrocytic tangles and neurites were found in the subcortical lesions [54]. Compared to Case 1, Case 2 had a lower extent of pTau accumulation in neurons and glia of the cortex and in secondary affected areas, including the cingulate gyrus and medial nucleus of the thalamus, but both cases had mild NFT Braak stage I (Case 1) and stage II (Case 2) in the hippocampus as observed with Gallyas-Braak staining [54]. Immunoblot analyses indicated that in both cases 3R and 4R tau isoforms accumulated along with pTau (pS396), which is observed in both AD and CTE due to rmTBIs.

In another case report, the authors investigated whether CTE tau pathology could occur after a single severe TBI. The subject was a male who in his early 20s suffered a gunshot wound to the head centered on the left frontal lobe from a 12-gauge shotgun, resulting in right-sided hemiplegia, epilepsy, and cognitive impairment [79]. He had no history of falls or other forms of TBI and died at age 63, 42 years post-injury, from multi-organ failure [79]. Post-mortem examination revealed pTau (AT8) in the medial temporal lobe entorhinal and transentorhinal regions, estimated to be Braak stage II neurofibrillary change [79]. Around the damaged tissue, pre-tangles (as defined by AT8 antibody staining) were observed focally within astrocytes and nearby neurons. In tissue away from the injured area, AT8 positivity was observed in perivascular, subpial, and subependymal areas [79]. This pattern, AT8 staining within neurons, astrocytes, and cell processes around blood vessels at the depths of cortical sulci in irregular foci, is the pathology observed in CTE due to repetitive TBIs [47, 79].

Kenney et al. reported the neuropathological findings from two patients who developed early-onset dementia after a msTBI. For the purposes of this review, data from patient 1 was not extracted since he suffered more than one TBI. Patient 2 was a female college graduate who suffered a severe TBI due to a motor vehicle accident at age 39 [35]. She was in a coma for 24 h and was in a post-traumatic confusional state with fluctuating levels of alertness, sleep disruption, and periods of agitation for an uncertain period post trauma, however, her posttraumatic amnesia resolved 6 weeks post-injury. Four to five years later she developed increased anxiety, headaches, depression, and progressive decline in memory and concentration [35]. Examined 12 years post-injury, she had significant deficits in executive function and semantic memory. These deficits were progressive and she died at age 63. Neuropathological examination revealed abundant NFTs and tau neurites in the dorsolateral and periventricular thalamus, hypothalamus, nucleus basalis of Meynert, mammillary bodies, brainstem tegmentum, substantia nigra, median raphe, and locus coeruleus [35]. NFTs and tau dot-like lesions were also observed, predominantly in the superficial layers of the cerebral cortex, deep gray and brainstem nuclei, and mamillary bodies [35]. CTE-like patterns of tau deposition were also observed in patient 2 with occasional perivascular AT8 pTau in thorned-shaped astrocytes and subpial regions [35]. Of note, patient 2 had a family history of early-onset AD and she was homozygous for ApoE4; therefore, her symptoms and pathology that developed post-severe TBI could have been accelerated from the head injury or by a synergistic effect with her genetic endowment.

Overall, the case studies included in this review suggest that after a single msTBI, tau pathology can be observed through PET imaging, pTau staining (as revealed by AT8 and pS422), and pTau immunoblots (with RD3, RD4, and pS396) and develops at later time points (from 2.4 years to over 53 years) and in a CTE-like pattern. In some of these studies, tau pathology was observed distal to the injury site, suggesting a potential spreading of tau like other tauopathies or could potentially stem from the trauma itself via rotational damage or contrecoup effects. These case studies, however, lack appropriate control groups or sufficient sample sizes to definitively ascertain if tau pathology develops chronically after msTBI or might interact with aging or other relevant factors, such as genetics and lifestyle; therefore, interpretation should be made cautiously.

### Animal studies

#### C57BL/6 mouse models

To deduce if a single severe TBI could induce self-propagating tau pathology, Zanier et al. randomly assigned male C57BL/6 J mice to receive a CCI brain injury over the left parietotemporal cortex, or sham injury, at 2mo of age. Tau pathology (with antibodies AT8, AT180, and PHF1) was then assessed at 3 or 12mo post-injury in both the ipsilateral and contralateral hemispheres. At 3mo post-injury, AT8 immunoreactivity was restricted to the ipsilateral cortex and regions adjacent to the injury, such as the hippocampus, in 2 out of the 4 TBI mice, but this was not statistically different from the 3mo sham mice that lacked AT8 positivity [88]. One year post-injury, AT8 immunoreactivity was found in both the ipsilateral and contralateral cortex, hippocampi, and thalamus in all the TBI mice (n = 4), which was significantly different from the sham mice at 12mo [88]. A similar pattern was observed with western blots, in which at 3mo post-injury 1 of the 4 TBI mice showed qualitative increases in total tau and AT8, while at 12mo, 2 of the remaining 3 TBI mice displayed qualitative increases in total tau and AT8 compared to shams [88]. These findings indicate that tau pathology after one severe TBI in mice progressively develops and propagates throughout the brain with age.

Kondo and colleagues targeted cis pTau, a toxic form of tau found early in pathology development [52], in mice that underwent a single severe TBI to determine whether it would mitigate “cis-tauosis”. “Cis-tauosis” was defined as the pathological process of cis pTau disrupting axonal microtubule networks and mitochondrial transport, spreading to other neurons, and inducing apoptosis [38]. Male C57BL/6 J mice 2–3mo of age were randomly assigned to undergo either a single severe TBI or sham injury over the dorsal aspect of the skull, using the weight drop injury model. TBI and sham mice were randomized to receive either an anti-cis pTau monoclonal mouse antibody or mouse IgG2b control treatment three days before injury and then 15 min post-injury followed by identical administration of the compounds three times every 4 days, then weekly for another 1.5 months (total of 2 months of treatment). Six months after TBI, the spread of cis pTau, tau aggregation and tauopathy were assayed. For the purposes of this review, we focused on the severe TBI mice that received the control treatment to address if the injury would lead to long-term development of tauopathy. Six months post-injury, TBI mice that received the IgG2b control treatment developed significantly more cis pTau and total tau (stained with tau-5) in the cortex and hippocampus and more insoluble tau aggregates, as well as more PHF1 (pS396/404), Alz50 staining (misfolded tau), AT8 (pS202/T205), and AT100 (pT212/S214) immunoreactivity compared to TBI mice that received the anti-cis pTau antibody treatment and sham mice. Thus, a single severe TBI leads to chronic tau aggregation and tau pathology, particularly the toxic cis pTau form [38].

Albayram et al. used the same study design as the Kondo et al. 2015 study, but only administering the treatment after the TBI (daily for 10 days, then weekly for another 1.5 months, then biweekly for another 2 months, for a total of 4 months of treatment). They found that 6mo post-injury, TBI mice that had received the IgG2b control treatment developed cis pTau, tau oligomers, and both early and late tau tangles (as revealed by AT8 and AT100 staining, respectively) [2]. These pathologies were found in both the medial prefrontal cortex and hippocampus. However, tau pathology in the TBI mice that received the anti-cis pTau antibody was eliminated and in the sham mice was not detectable [2]. These results support those found in the Kondo et al. 2015 study [38].

Male C57BL6/SV129 and Tau knockout mice (Tau^−/−^) received either a TBI using the FPI model or a sham injury to deduce if tau reduction would affect pathology after a single msTBI [77]. We extracted only the outcomes from the C57BL6/SV129 mice. Twelve weeks post-injury, TBI resulted in a significantly higher pS198/tau5 ratio in the injured cortex compared to sham mice, but there were no significant differences in the pS398/tau5 ratio or in total tau levels [77]. These findings suggest that chronically post-TBI, phosphorylated epitopes of tau are differentially affected.

Taken together, TBI studies utilizing C57BL/6 mice with different injury models (1 study using CCI, 2 using weight drop, and 1 employing FPI) demonstrated that a single msTBI leads to development of mouse tau pathology, including pTau, misfolded tau, and insoluble aggregated tau, at either 3mo, 6mo, or 12mo post-injury. Since only one study of the four assessed the presence or absence of tau pathology in the contralateral hemisphere [88], from these results we can only deduce that a single msTBI leads to long-term development of tau pathology, but not necessarily any spread or self-propagation of tau pathology from the injury site. In addition, more widespread dissemination of tau pathology may require longer times to manifest in the other hemisphere.

#### Rat models

Glushakova et al. (2018) evaluated changes in cleaved-caspase-3 and caspase-3-cleaved tau truncated at Asp421 and their associations with inflammation and blood–brain barrier damage in the white matter post TBI. For our review, only data on caspase-3-cleaved tau in the white matter post TBI was extracted. In this study, male Sprague–Dawley rats were randomly assigned to receive either a single moderate to severe CCI or sham injury over the right parietal region. Three months post-CCI, there was a significant upregulation in caspase-3-cleaved truncated tau in the corpus callosum aggregating around blood vessels, compared to sham injured controls [24]. Truncated tau was also observed both extracellularly and intracellularly around cell nuclei or dead cells [24]. These results suggest that a single msTBI upregulates truncated tau that could contribute to changes in white matter post-injury.

The primary objective of Shultz et al. (2015) was to investigate if a single severe TBI affected protein phosphatase 2A (PP2A), a major tau phosphatase in the brain, and pTau, and then whether treatment with sodium selenate, an activator of the subunit PR55 on PP2A, would reduce pTau and improve outcomes post-TBI [69]. Male Long-Evans rats 12 weeks of age were randomly assigned to receive FPI or sham injury positioned -3 mm posterior and 4 mm lateral of bregma. Mice were then randomly assigned to receive either saline-vehicle or sodium selenate treatment continuously (via subcutaneous osmotic pump 1 mg/kg/day) for the duration of the study (12 weeks post-injury). At 12 weeks post-injury, FPI + saline vehicle treated rats had significantly increased pS198/total tau ratio, but the ratio of pS262/total tau in the injured cortex was not changed, compared to sham + saline vehicle treated rats [69]. The expression levels of pS198 and pS262 were not significantly different between the groups, but total tau was significantly reduced in the FPI groups regardless of treatment, compared to sham-injured rats. This may indicate that the pS198/total tau ratio was increased because of the significant reduction in total tau levels of injured rats compared to sham rats [69]. These results suggest that a single severe TBI leads to chronic decreased total tau levels and may affect specific tau phosphorylation epitopes.

Seeking to determine possible AD-like pathology in the chronic (6 months) phase post-TBI, Acosta et al. (2017) subjected 2-month-old Sprague–Dawley rats to either a CCI or sham injury over the lateral right fronto-parietal cortex. Six months later, rats that received a TBI had significantly more AT8 + immunostaining in the ipsilateral frontal cortex and dentate gyrus of the hippocampus, but not in the contralateral hemispheres, compared to sham control rats [1]. T22 + immunostaining, a marker for oligomeric tau, was increased significantly in TBI rats compared to sham control rats in both the ipsilateral and contralateral cortex and dentate gyrus of the hippocampus [1]. These data suggest that hyperphosphorylated tau and oligomeric tau are present long-term following TBI and that certain tau species (such as oligomers) can spread to the contralateral side, likely via trans-synaptic axonal pathways [42].

In sum, these rat studies also indicate that a single msTBI leads to long-term (either 3 months or 6 months) deposition and maintenance of hyperphosphorylated, truncated, and oligomeric tau, albeit in different distributions. They also suggest an increase in specific epitopes of hyperphosphorylated tau post-TBI, and also that total tau might be reduced (only 1 of the 3 studies assessed total tau). Since only one study assessed the contralateral hemisphere for the spread of tau pathology, we cannot address fully if a single msTBI leads to the spread of tau pathology, which would also require longer term studies [1].

#### Transgenic mouse models

In a somewhat similar design in transgenic mice, Edwards et al. sought to determine if a msTBI could trigger pathological tau that could propagate throughout the brain. They utilized the P301S (PS19) transgenic mouse model of tauopathy; these mice express human tau (4R/1 N tau isoform) containing the P301S mutation, which is associated with early-onset frontotemporal dementia with parkinsonism linked to chromosome 17 (FTDP-17) [72]. Human tau in the PS19 model is fivefold higher than endogenous mouse tau, and these mice develop age-dependent tau pathology beginning around 3mo [86]. Three-month-old PS19 mice were subjected to either a CCI or sham-injury to the right parietal cortex. Six months later, TBI-treated mice manifested NFTs throughout the ERC and hippocampus and had significantly more widespread and increased AT8 tau compared to sham controls in the ipsilateral and contralateral cortical and hippocampal areas [21]. Tau levels were significantly augmented compared to sham controls in the amygdala, piriform/ERC, and brainstem [21]. Wild type (WT) mice in the same paradigm had no AT8 staining 6mo post-injury [21]. Thus, 6mo post single msTBI, hyperphosphorylated tau was significantly upregulated and spread to the contralateral hemisphere. These results should be interpreted with some caution because the sham group did not go through all the same procedures as the TBI mice. Sham mice did not receive a craniotomy like the TBI group, meaning that the increased phosphorylation observed in the TBI group could be confounded by increased anesthesia time and vibratory effects on the underlying cortex during the procedure.

Kokiko-Cochran et al. (2018) characterized the microglial/macrophage response to TBI and secondarily assessed tau pathology using hTau (human tau) mice, which contain all WT human tau 3R and 4R isoforms on a genetic mouse tau knockout background [3, 18]. These mice develop age-dependent tau pathology beginning at 6mo [3, 18]. Male and female hTau and WT mice received either a moderate lateral FPI or sham injury over the right parietal cortex at 2mo. Around 4mo post-injury, hTau TBI mice had significantly more AT180 (pT231) tau immunoreactivity in the injured cortex compared to hTau sham and WT TBI and sham groups [37]. In the ipsilateral temporal cortex, hTau TBI mice did not manifest AT180 tau staining that was significantly different from the hTau sham mice, but the staining was significantly different from WT TBI and WT sham mice, indicating that post-TBI changes in pTau are likely region-specific in hTau mice [37]. In addition, hTau TBI mice exhibited accelerated Gallyas positive silver staining, indicating mature NFTs, in the ipsilateral hippocampus 4mo post-TBI, which typically does not appear in uninjured hTau mice until 9mo of age [3, 37]. There were no differences in Gallyas silver staining in the contralateral hippocampus. These data suggest that several months post msTBI, there are region-specific increases in pTau and acceleration of the development of NFTs.

Overall, regardless of the injury model used (transgenic mouse models with either human WT tau or human mutant tau linked to FTDP-17), the single msTBI studies demonstrated that at (relatively) chronic time points (4mo or 6mo) after rodent injury, there were 1) increased and accelerated tau pathology, 2) increased pTau deposition, and 3) increased tau pathology, all in a region-specific manner.

## Discussion

MsTBIs increase an individual’s risk for developing dementia by 2–fourfold compared to no history of head injury, and may contribute to 5–15% of all dementia [67]. The pathology that develops post-TBI is controversial, as many neurodegenerative disease-associated proteins accumulate in traumatized brains [80]. Some investigators speculate that the tau pathology observed in human TBI can be attributed not to the injury itself, but to primary age-related tauopathy (PART), an age-associated accumulation of tau pathology restricted mainly to the hippocampus [15]. CTE, a neurodegenerative disease linked to rmTBIs, is defined pathologically by abnormally hyperphosphorylated tau within neurons and astrocytes at the depths of cortical sulci and around blood vessels, in irregular and patchy distributions [44]. Many speculate that msTBI can lead to tau pathology like that observed in CTE. Based on both the human and preclinical animal studies in this review, msTBI leads to the long-term development of tau pathology, including hyperphosphorylated, truncated, and misfolded tau, the appearance of epitope-specific tau pathology on a time and region-specific manner, and deposition of tau pathology in some individuals in a similar pattern to that observed in CTE.

Of the 15 observational human studies, 12 studies concluded that long-term survival post msTBI leads to the development of tau pathology, while only 3 studies concluded that it does not lead to chronic development of abnormal tau. These 3 studies should be interpreted with caution, though, as they had certain limitations [6, 23, 40]. First, these studies relied on blood or CSF total tau rather than pathology. Total tau increases following cell damage and, while total tau can be elevated in tauopathies, this elevation is correlated more with acute injury and/or rate of on-going neurodegeneration rather than being specific to tau pathological burden [11, 29, 63]. Further, in Franz et al. (2003), the two control groups were either individuals who had cognitive disorders (a dementia group, which included patients with AD, vascular dementia, frontotemporal dementia, and normal pressure hydrocephalus), or a second control group, comprised of individuals with headache [23]. Tau pathology is frequent in dementia; thus, it might not be the best control to deduce if a msTBI can lead to long-term development of tau pathology [32]. It might be useful in description of different patterns of tau distribution, however. In this study, the authors only assessed total tau in the CSF, which missed opportunities to assess other changes in tau that are often observed in disease, such as changes in truncation or phosphorylation of tau. Further, CSF was collected for the TBI and control groups with different methods. Fifteen of the TBI patients had CSF collected from intraventricular catheters, while the remaining 14 TBI patients and all controls had CSF collected from lumbar puncture [23]. Collecting CSF from different compartments along the neuraxis can introduce variability, e.g., varying amounts of tau levels per compartment. It is known that protein concentration in the CSF increases in a rostrocaudal gradient, with ventricular protein concentration one third of that in the lumbar space [9]. Further, the TBI group sustained different initial injuries, with some having to undergo craniotomy due to epidural or subdural hematoma. The possibility of these procedures producing variable tau levels was not addressed. In Bagnato et al. (2018) the authors assessed total tau and pTau (pThr181) in individuals who experienced prolonged loss of consciousness post-TBI, but this patient population is a specific subset of TBI individuals and is not representative of the broad TBI population. Finally, this study did not include controls, only referencing normalized tau values taken from a group of healthy controls from a separate study which did not indicate if they had or did not have a history of TBI [71, 81]. Bagnato et al. (2018), also did not divide their TBI group into short vs. long-term survival; 7 of the 15 patients had less than 6 months since their TBI [6]. Because the time from TBI was in our target range for the review, we included this study, but individuals with less than 6 month survival could have skewed the results to no significant difference; this fact should be considered. Lekomsteva et al. (2020) concluded that total tau in the serum was not increased significantly in moderate TBI compared to healthy controls, but there was considerable heterogeneity in the patient population; 7% of the patients were experiencing 1 to 2 seizures per month and taking an antiepileptic drug, 47.6% had a mild neurological deficit, and one patient had severe facial sympathalgia and was being treated with two analgesics [40]. There was no statement that the controls had no history of TBI, only that they had no known neurological, psychiatric, or somatic pathology [40]. This study also did not assess pTau or other tau abnormalities that could develop chronically.

All other human articles included in this review reported that a msTBI does lead to the long-term development of tau pathology, including hyperphosphorylated tau, oligomeric tau, truncated tau, misfolded conformational tau, and NFTs. Further, the tau pathology that developed following msTBI was similar to pathology observed in CTE and distinct from pathology in PART [44]. Involvement of both 3R and 4R tau was observed in msTBI patients, similar to AD and other tauopathies [32]. Further, some studies discovered that tau pathology was accelerated or appeared at ages as young as 27 years old compared to controls, potentially indicating that the pathology is not strictly associated with aging. Interestingly, tau pathology that did develop chronically post-TBI appeared to be both time and region dependent. Some studies that assessed tau longitudinally found that at 1 and 2 years post-TBI, total tau was increased in serum samples, but this was not seen at longer time points, such as at 3–5 years post-TBI [66]. For development of therapeutic interventions, targeting specific tau changes must be defined for specific time points throughout the course of disease. Subsequent studies will be needed to fully elucidate how such timed interventions might mitigate pathology and cognitive progression. Further, studies that assessed tau pathology in different brain regions determined that pathology was increased in certain regions, decreased in others, or was unchanged from controls. Regional vulnerability to tau pathology could be due to injury location(s) and/or genetic responses in different brain regions. Similar to Braak Staging in AD, the regional spread of pTau in CTE due from rmTBIs has been assessed and grouped into four stages: Stage I, pTau is restricted to the cerebral cortex, mainly the frontal cortices, as discrete foci at the depths of sulci and around vessels; Stage II, pTau spreads into adjacent cortical regions forming multiple epicenters; Stage III/IV, pTau is more widespread and severely effects the frontal and temporal lobes [48]. Staging the regional spread of pTau due from msTBI analogous to Braak and CTE Staging, has not been conducted. Some human studies included in this review compared the pTau severity to that observed in Braak Staging, but a full staging schematic is still lacking [49, 54, 79]. Due to the heterogeneity of where pTau was observed and which brain regions were sampled in studies included in this review, this was beyond the scope for this review, but will be critical to assess in future studies. Further, not every individual who sustained a msTBI developed chronic tau pathology; Johnson et al. (2012) found that only ~ 1/3 of their cases manifested NFTs in the long-term period post-TBI [34]. Thus, in addition to the variability in severity and nature of TBI, genetic contributions to the susceptibility and resistance to tau pathology will need investigation. In addition to genetic variability, differences in type of injuries, such as whether it involved a craniotomy, intracerebral hemorrhage, leucotomy, presence of seizures, etc., that could affect tau pathology must be further determined.

Of course, interpreting results from human studies comes with significant limitations. Most of the human studies involved only males, leaving a significant knowledge gap of how females respond long-term post msTBI. Future studies will need to address post-TBI tau pathology in females, who may have a higher susceptibility to TBI [10, 14, 17, 85]. Additional factors, such as genetics, ethnicity, socioeconomic status, and educational level, will be important to assess for how they may influence tau pathology. The presence of the APOE e4 allele, for example, has been associated with longer recovery time after injury and may contribute to further tau pathology [51, 57, 89]. Assessing the brain location of where the injury was sustained in TBI patients was not considered, but may help explain regional vulnerabilities to the development of tau pathology. Finally, investigating the spread of tau pathology from one region to another was not conducted. The case studies included in this review, although with no comparators, helped shed potential light on the spread of tau pathology from the site of injury to other brain regions. Two of the case studies reported the presence of tau pathology distal to the injury site many years after trauma, suggesting that the injuries either promoted tau spread after msTBI or were caused from contrecoup effects [54, 79]. Future studies will need to explore this phenomenon in both animals and humans.

Preclinical animal studies demonstrated that a msTBI leads to long-term development of tau pathology, including pTau, oligomeric tau, truncated tau, misfolded tau, and NFTs, similar to what has been observed in the human studies. Some studies assessed the same pTau epitope and time since TBI and produced consistent results. AT8, which assess pTau at Serine202, was significantly increased compared to sham animals as early as 3mo post-injury in different rodents–C57BL/6 J mice, P301S mice, and Sprague–Dawley rats—and with different injury models (CCI or weight drop) [1, 2, 21, 38, 88]. With total tau, results were not as consistent across the different injury models and time post-injury. Some studies observed increases in total tau at 6 or 12mo post-TBI using either the CCI or weight drop models of injury [38, 88], while others found decreases in total tau or stable levels in total tau 12 weeks post-TBI using the FPI model [69, 77]. Thus, at early time points post-TBI, total tau may not be affected (or may not yet be detectable by current techniques), only manifesting at longer intervals. Two studies assessing cis pTau and AT100 both observed cis pTau and AT100 in TBI compared to unexposed or sham mice using the weight drop injury model [2, 38]. Assessment of PHF1 tau or pS396 tau, found conflicting results [38, 77]. Kondo et al. (2015) found an increase in PHF1 tau 6 months post-injury with the weight drop model, while Tan et al. (2020) found no difference at 12 weeks post-injury using the FPI model [38, 77]. This might imply that pathological alterations at this specific phosphorylation site may be time-dependent and evolve at longer time points, or that the different injury models used in these studies induce variability. Two studies assessing pS198 both found increases 12 weeks post-TBI using the FPI model [69, 77]. Oligomeric tau was also significantly increased 6 months post-TBI in two studies that used either the weight drop or CCI model [1, 2]. Thus, post msTBI, specific tau epitopes may become more vulnerable to phosphorylation changes at specific time points in progression of pathology. Some, such as pS198, may change only at early time points post-TBI, while PHF1 may evolve more slowly, and AT8 is manifested continuously during the pathological progression. This pattern of tau phosphorylation at specific time points in pathology progression post-TBI has been observed in AD, albeit with different residues affected [5, 53].

Although all preclinical animal studies included in this review resulted in long-term development of tau pathology post msTBI, limitations to the studies should be considered. In all of the studies except two, only males were used, with no justification for exclusion of females. It is important to incorporate females into studies, though, as underlying mechanisms post TBI may well be different between the sexes. Understanding how both sexes respond to injuries will aid therapeutic development, especially if there are sex differences in response to therapeutics. It would also help if injury models in preclinical animal studies were standardized. Even when using the same injury model, the injury parameters were vastly different in some studies. Both Zanier et al. and Edwards et al. used the CCI injury model, but one group maintained stable rectal temperatures during anesthesia and surgery, while the other group did not. Anesthesia promotes tau phosphorylation via anesthesia-induced hypothermia, but maintaining core body temperature during the procedure can prevent this [60, 82]. Having temperature control integrated into injury protocols and specifying how long anesthesia was delivered should be reported, to help reduce variability and increase consistency. Further, defining the specific injury parameters, such as impact velocities, penetration depth, etc., that define different levels of injury severity per injury model is also needed. In the animal studies in this review, different injury parameters were used in the same injury model, but were defined as having the same injury severity; the manuscripts did not always report how they defined those injury parameters. Most studies in this review did not conduct a power analysis and the small sample sizes may well have affected the results. Finally, some studies only assessed one tau hyperphosphorylation epitope, missing an opportunity to assess differential pathology development and other changes in tau post-TBI.

The scope of this review was to assess if chronic tau pathology developed post msTBI, but there were limitations. We did not assess if tau pathology correlated with cognitive decline observed in TBI patients, which has been demonstrated with other diseases, such as AD [8]. This review also did not assess inflammation and how that may have affected the development of tau pathology. Previous TBI studies found that inflammation persisted long after injury and that inflammation can influence tau pathology and outcomes post TBI [25, 36, 70]. Although our purpose was to assess long-term outcomes post msTBI, earlier time points post-injury would be beneficial to investigate, to determine potential early biomarker indicators. Knowledge of early changes in tau combined with chronic and persistent abnormalities in tau can help physicians identify and intervene in those at risk for developing degenerative disease, and apply appropriate interventions.

## Conclusions

In summary, in both human and preclinical animal studies, we conclude that msTBI can lead to the long-term development of varying tau pathology, including hyperphosphorylated, truncated, misfolded, and oligomeric tau, as well as NFTs. In humans, some tau pathology appeared similar to that observed in CTE- within neurons and astrocytes at the depths of cortical sulci and around blood vessels in an irregular and patchy distribution [44]. In preclinical animal studies, the spread of tau pathology from the site of injury to the contralateral cortex suggested that TBI-induced pathological tau may spread in a prion-like fashion [88]. Thus, tau pathology is not transient after msTBI, but develops and remains in the brain tissue for varying periods of time, and possibly contributes to the development or progression of tauopathies in humans. Future research will investigate targeting tau post-injury to see if the intervention can slow or reverse tau pathology progression. Genetic contributions to the development of tau pathology in animal studies could be assessed utilizing specific transgenic animal models, such as those that contain different human APOE alleles or other modified proteins that accumulate in post-mortem traumatized human brains [80]. The paucity of TBI studies using transgenic animals leaves both a gap and an opportunity in the field for investigating long-term effects post msTBI. Incorporation of both sexes in such studies is also necessary, as well as developing a staging scheme for pTau spread due to msTBI. Such future studies will bring deeper insight into the pathogenesis induced by msTBI and help identify targets for therapeutic development.

## Supplementary Information

**Additional file 1**: This table discloses of the study characteristics for human based articles, including article title, study design, injury severity, injury rating, injury type, sample size, age at time of study for TBI and control populations, inclusion and exclusion criteria, post-TBI interval (time since injury), type of tau assessment, findings, and if those findings supported chronic tau development.

**Additional file 2**: This table discloses of the study characteristics for human based articles, including article title, study design, injury severity, injury rating, injury type, sample size, age at time of study for TBI and control populations, inclusion and exclusion criteria, post-TBI interval (time since injury), type of tau assessment, findings, and if those findings supported chronic tau development.

**Additional file 3**: This table discloses of the study characteristics for human based articles, including article title, study design, injury severity, injury rating, injury type, sample size, age at time of study for TBI and control populations, inclusion and exclusion criteria, post-TBI interval (time since injury), type of tau assessment, findings, and if those findings supported chronic tau development.

**Additional file 4**: This table discloses of the study characteristics for human based articles, including article title, study design, injury severity, injury rating, injury type, sample size, age at time of study for TBI and control populations, inclusion and exclusion criteria, post-TBI interval (time since injury), type of tau assessment, findings, and if those findings supported chronic tau development.

**Additional file 5**: This table discloses of the study characteristics for preclinical animal based articles, including article title, animal model, injury severity, injury rating, injury model, injury parameters, sample size, age of injury, post-TBI interval (time since injury), type of tau assessment, findings, and if those findings supported chronic tau development.

**Additional file 6**: This table discloses of the study characteristics for preclinical animal basedarticles, including article title, animal model, injury severity, injury rating, injury model, injuryparameters, sample size, age of injury, post-TBI interval (time since injury), type of tauassessment, findings, and if those findings supported chronic tau development.

## Data Availability

All data generated or analyzed during this study are included in this published article and its supplementary information files (included in the tables found in supplementary additional files 1–6).

## Abbreviations

3R: Three C-terminal repeats
4R: Four C-terminal repeats
4R/1 N: Four C-terminal repeats, one N-terminal repeat
Aβ: Amyloid beta
AD: Alzheimer’s Disease
ARTAG: Aging-related tau astrogliopathy
CBD: Cortical basal degeneration
CCI: Controlled cortical impact
CSF: Cerebrospinal fluid
CTE: Chronic traumatic encephalopathy
DTI: Diffusion tensor imaging
DVR: Distribution volume ratio
FPI: Fluid percussion injury
FTDP-17: Frontotemporal dementia with parkinsonism linked to chromosome 17
FTLD-tau: Frontotemporal lobar degeneration with tau inclusions
hTau: Human tau
mo: Months
MRI: Magnetic resonance imaging
msTBI: Moderate to severe TBI
NFTs: Neurofibrillary tangles
NOS: Newcastle–Ottawa Scale
PART: Primary age-related tauopathy
PCS: Post-concussive syndrome
PET: Positron emission tomography
PP2A: Protein phosphatase 2A
PRISMA: Preferred reporting items for systematic reviews and meta-analysis
PSP: Progressive supranuclear palsy
pTau: Phosphorylated tau
PTSD: Post-traumatic stress disorder
rmTBI: Repetitive mild traumatic brain injury
scFvs: Single chain variable fragments
TBI: Traumatic Brain Injury
WT: Wild-type
