# Supplementary material for: Association between single moderate to severe traumatic brain injury and long-term tauopathy in humans and preclinical animal models: a systematic narrative review of the literature
Source: Acta Neuropathol Commun. 2022 Jan 31;10:13. doi: 10.1186/s40478-022-01311-0 (PMC8805270; doi:10.1186/s40478-022-01311-0)
Supplement: Supplementary file 1 — Additional file 1: This table discloses of the study characteristics for human based articles, including article title, study design, injury severity, injury rating, injury type, sample size, age at time of study for TBI and control populations, inclusion and exclusion criteria, post-TBI interval (time since injury), type of tau assessment, findings, and if those findings supported chronic tau development. [file 40478_2022_1311_MOESM1_ESM.pdf]

**Table 1. Human Study Characteristics.**

| Article | Study Design | Injury Severity | Injury Rating | Injury Type | Sample Size ($n_{males}$) | Age (years) | Inclusion Criteria | Exclusion Criteria | Post-TBI Interval | Type of Tau Assessment | Findings | YES or NO Chronic Tau |
|---|---|---|---|---|---|---|---|---|---|---|---|---|
| Zanier et al. 2018 | Retrospective Cohort Study | Single moderate or severe | Glasgow Coma Scale | Road traffic accident n=5<br>Fall n=5<br>Assault n= 3<br>Not known n=2 | TBI n= 15 (12)<br>Controls n=15 (11) | TBI= 60 (median), 19-89 (range)<br>Controls= 60 (median), 20-92 (range) | NS | NS | 1-18 years | IHC using PHF1 | Proportion of PHF1 in TBI and control cases were similar (TBI=12 cases, Controls=12 cases), but the extent and distribution of PHF1 tau was greater in TBI (9 of 12) vs. controls (4 of 12) and appeared at younger ages in TBI (59yr+) compared to controls (79yr+) | YES |
| Franz et al. 2003 | Retrospective Cohort Study | Single severe | Glasgow Coma Scale | NS | TBI n=29 (27)<br>Cognitive Disorders Controls n= 19 (NS)<br>Headache Controls n= 12 (NS) | TBI= 41 (median), 15-72 (range)<br>Controls= NS | NS | NS | 1-284 days | Sandwich ELISA on total tau from CSF samples | Chronic time points post-TBI (43 days and greater) total tau levels were normal (median 153 pg/mL) | NO |
| Shahim et al. 2020 | Prospective Cohort Study | Single moderate to severe | Clinical history and criteria of Department of Defense and Veterans Affairs | Acceleration/deceleration<br>Blast<br>Direct impact/blow to head<br>Fall<br>Other | TBI n=73 (NS for injury severity)<br>Controls n= 68 (NS for injury severity) | TBI= 43 (median), 30-56 (IQR)<br>Controls= 42 (median), 27-54 (IQR) | TBI: (1) male or female >18 years of age; (2) clinical diagnosis of nonpenetrating TBI, and (3) injury occurring <1 year before enrollment<br>Control: (1) ≥18 years of age, (2) good general medical and psychological health based on history and physical examination by licensed medical staff, (3) no history of heavy alcohol use or substance abuse, and (4) no history of head injury, regardless of cause | TBI: (1) contraindications to MRI, including foreign metallic objects and noncompatible metallic devices or objects; (2) a history of major neurologic or psychiatric conditions such as multiple sclerosis, stroke, spinal cord injury, or psychosis; and (3) pregnancy<br>Control: NS | 30-180 days and 1-5 years | Neurology 4-Plex Assay kit on total tau from serum samples | Single severe TBI had significantly more serum total tau at 1 and 2 years post injury vs. controls, but not at the 3-5 year timepoints | YES |
| Mohamed et al. 2018 | Multimodality non-randomized natural history non-intervention Cohort Study | Single moderate to severe | Well documented history of head trauma using military and Veterans Affairs Compensation and Pension records | Military service from serving in Vietnam War | TBI n=9 (9)<br>Controls= 27 (27) | TBI= 67.9 (mean), 4.4 (SD)<br>Controls= 70.9 (mean), 6.01 (SD) | TBI: Subjects must be Veterans of the Vietnam War, 50-90 years of age. Subjects must have a documented history of moderate-severe non-penetrating TBI, which occurred during military service in Vietnam. Must live within 150 miles of the closest ADNI clinic in subject's area.<br>Control: Subjects must be Veterans of the Vietnam War, 50-90 years of age, comparable in age, gender, and education with TBI and PTSD groups, may be receiving VA disability payments for something other than TBI or PTSD – or no disability at all. Must live within 150 miles of the closest ADNI clinic in subject's area. | TBI: Mild Cognitive Impairment/Dementia Presence of PTSD by SCID-I for DSM-IV-TR criteria, or a CAPS score of >30 (Both current and/or a history of PTSD will be excluded).<br>Control: MCI/Dementia Presence of PTSD by SCID-I for DSM-IV-TR criteria, or a CAPS score of >30 (Both current and/or a history of PTSD will be excluded). Documented or self report history of mild/moderate severe TBI. Any history of head trauma associated with injury onset cognitive complaints, or Loss of Consciousness for >5 minutes, History of PTSD or current PTSD | NS* | Luminex xMAP multiplex immunoassay on total tau and p-tau (Thr181) from CSF samples | In TBI group, amyloid burden in the brain significantly correlated positively with CSF tau in the frontal ($r^2$= 0.36), cingulate ($r^2$= 0.3), parietal ($r^2$= 0.33), and temporal cortex ($r^2$= 0.3) vs. healthy controls which displayed a positive correlation only in the frontal ($r^2$= 0.16) and temporal cortex ($r^2$= 0.24) | YES |

NS= not stated, IQR= interquartile range, SD= standard deviation, *= did not disclose but since patients served in Vietnam War and head injury had to have occurred during service this meets criteria
