# Supplementary material for: Association between single moderate to severe traumatic brain injury and long-term tauopathy in humans and preclinical animal models: a systematic narrative review of the literature
Source: Acta Neuropathol Commun. 2022 Jan 31;10:13. doi: 10.1186/s40478-022-01311-0 (PMC8805270; doi:10.1186/s40478-022-01311-0)
Supplement: Supplementary file 2 — Additional file 2: This table discloses of the study characteristics for human based articles, including article title, study design, injury severity, injury rating, injury type, sample size, age at time of study for TBI and control populations, inclusion and exclusion criteria, post-TBI interval (time since injury), type of tau assessment, findings, and if those findings supported chronic tau development. [file 40478_2022_1311_MOESM2_ESM.pdf]

Table 1 cont. Human Study Characteristics.

Article	Study Design	Injury Severity	Injury Rating	Injury Type	Sample Size (n_{males})	Age (years)	Inclusion Criteria	Exclusion Criteria	Post-TBI Interval	Type of Tau Assessment	Findings	YES or NO Chronic Tau
Bagnato et al. 2018	Retrospective Cohort Study	Single severe	NS	NS	TBI n=15 (14)<br>No Controls	TBI= 32.1 (mean), 13.1 (SD)	(1) diagnosis of unresponsive wakefulness syndrome (UWS) or minimally conscious state (MCS) after a TBI at the time of lumbar puncture execution, (2) a TBI-to-lumbar puncture interval greater than 90 days.	Patients with a history of prior neurological diseases were not included nor were patients over 60 years old due to the risk trauma-independent a priori AD pathology; patients were not included if a CSF tap test indicated an improvement in their level of consciousness or if their CSF analysis revealed a current CNS infection.	92-578 days	ELISA on total tau and p-tau (Thr181)	Chronic phase post-TBI does not lead to abnormal total tau in all severe TBI cases (median 95.2 pg/ml, range 52-256.9 pg/ml) or p-tau (Thr181) in 14 of the 15 severe TBI cases (median 22.2 pg/ml, range 14-72 pg/ml) that developed prolonged posttraumatic disorders of consciousness compared to reference normal values (<300 pg/ml for total tau and <61 pg/ml for p-tau)	NO
Takahata et al. 2019	Retrospective Cohort Study	Single severe	Severe TBI defined as head injury resulting in loss of consciousness >24hr (Glasgow Coma Scale)	Traffic accidents n=2<br>Falls n=12	TBI n=14 (12)<br>Controls n= 15 (10)	TBI= 43.2 (mean), 13.6 (SD)<br>Controls= 43.4 (mean), 14.4 (SD)	TBI: (1) patients with a history of either severe TBI or mild-repetitive TBI, (2) aged 20 years and older, (3) absence of any neuropsychiatric or neurological disorders prior to or within 1 year of the head injury; and (4) absence of severe physical diseases<br>Control: healthy subjects without a history of TBI or other neurological or psychiatric diseases	NS	2.4- 26.6 years	PET imaging using C-PBB3	C-PBB3 tau binding capacity was significantly higher in the neocortical grey matter (temporal and occipital) in TBI (median 16.5 ± 7.5 cm^3) vs. healthy controls (median 10.2 ± 4.6 cm^3), white matter (temporal, frontal, and occipital) in TBI (median 14.2 ± 5.4 cm^3) vs. healthy controls (median 9.4 ± 3.8 cm^3), and in the medial frontal white matter and surface of the white matter in TBI vs healthy controls	YES
Mohamed et al. 2019	Retrospective (multimodal non-randomized) Cohort Study	Single moderate to severe non-penetrating	Well documented history of head trauma using military and Veterans Affairs Compensation and Pension records	Military service from serving in Vietnam War	TBI n=10 (10)<br>Controls n=21 (21)	TBI= 72.6 (mean), 6.82 (SD)<br>Controls= 74.29 (mean), 7.2 (SD)	Refer to Mohamed et al. 2018	Refer to Mohamed et al. 2018	NS*	PET imaging using [18F]AV1451	TBI group had significantly higher mean SUVr in frontal lobe (1.06 ± 0.05) compared to healthy controls (1 ± 0.07), but significantly lower tau uptake in the left inferior temporal gyrus (1.21 ± 0.16) vs. controls (1.25 ± 0.15)	YES
Wooten et al. 2019	Retrospective Cohort Study	Single severe	NS	Automotive accident	TBI n=1 (1)<br>Controls n=11 (9)	TBI= 34<br>Controls= 47 (mean), 26-67 (range)	Controls must have no self-reported history of TBI	NS	2.5 years	PET imaging using [18F]AV1451	The single severe injury subject had highest tau binding in corpus callosum, thalamus, and brainstem compared to all other subjects	YES
Shively et al. 2017	Retrospective Cohort Study	Single severe	NS	Surgical leucotomy	Schizophrenia Surgical Leucotomy n= 5 (2)<br>Schizophrenia Control non-leucotomized n=5 (2)	Leucotomized= 78 (mean), 67-89 (range)<br>Non-Leucotomized= 76.6 (mean), 67-86 (range)	NS	NS	~40 years	IHC using AT8, CP13, PHF1, and Bielschowsky silver stain	All five leucotomy cases revealed NFTs and p-tau neurites in overlying gray matter of leucotomized site and tau pathology could be observed in areas rostral and caudal to the leucotomized site including hippocampus of some leucotomy cases v. non-leucotomized patients; CTE-like pattern of tau pathology also observed in majority of leucotomy cases v. non-leucotomized controls	YES

NS= not stated, SD= standard deviation, *= did not disclose but since patients served in Vietnam War and head injury had to have occurred during service this meets criteria
