# Supplementary material for: Association between single moderate to severe traumatic brain injury and long-term tauopathy in humans and preclinical animal models: a systematic narrative review of the literature
Source: Acta Neuropathol Commun. 2022 Jan 31;10:13. doi: 10.1186/s40478-022-01311-0 (PMC8805270; doi:10.1186/s40478-022-01311-0)
Supplement: Supplementary file 3 — Additional file 3: This table discloses of the study characteristics for human based articles, including article title, study design, injury severity, injury rating, injury type, sample size, age at time of study for TBI and control populations, inclusion and exclusion criteria, post-TBI interval (time since injury), type of tau assessment, findings, and if those findings supported chronic tau development. [file 40478_2022_1311_MOESM3_ESM.pdf]

Table 1 cont. Human Study Characteristics.

Article	Study Design	Injury Severity	Injury Rating	Injury Type	Sample Size (n_{males})	Age (years)	Inclusion Criteria	Exclusion Criteria	Post-TBI Interval	Type of Tau Assessment	Findings	YES or NO Chronic Tau
Lekomtseva et al. 2020	Retrospective Cohort Study	Single moderate	International Statistical Classification of Diseases and Related Health Problems; Glasgow Coma Scale	NS	TBI n=42 (31)<br>Controls n=30 (NS)	TBI= 37.36 (mean), 10.2 (SD)<br>Controls= 29.6 (mean), 4.73 (SD)	Controls without known neurological, psychiatric, and somatic pathology	For all groups: craniotomy and sepsis in their anamnesis, pregnancy, preexisting neurologic or psychiatric diseases, acute or chronic cardiovascular diseases, respiratory failure, acute and chronic liver diseases	12 months	ELISA on total tau in serum	No significant difference in total tau concentration in moderate TBI (82.27 ± 26.37 pg/ml) vs. controls (71.14 ± 20.56 pg/ml) but there was a "trend" in tau concentrations being higher in moderate TBI vs. controls	NO
Rubenstein et al. 2015	Retrospective Cohort Study	Single severe	Glasgow Coma Scale	Blunt trauma to head	TBI n=4 (NS)<br>Controls= 4 (NS)	TBI= NS<br>Controls= NS	NS	NS	6 months	Novel immunoassay EIMAF on total tau (DA31) and p-tau (RZ3) from serum	Serum p-tau, but not total tau, was higher in single severe TBI vs. controls	YES
Johnson et al. 2012	Retrospective Cohort Study	Single moderate to severe	NS	Assault n=8<br>Fall n=16<br>Motor vehicle accident n=8<br>Unknown n=7	TBI n=39 (35)<br>Controls n=47 (30)	TBI= 53 (mean), 19-89 (range)<br>Controls= 47 (mean), 14-92 (range)	Control: age-matched with no history or confirmed history of TBI and no neurodegenerative disease or neurological disease	TBI: history of amateur or professional boxing	1-47 years	Immunostaining using tau polyclonal antibody and thioflavin-S	~22% of TBI cases contained extensive and widespread NFTs compared to controls (~6%) and ~1/3 of TBI participants ≤60 years had significantly more NFTs (11 of 32) compared to controls ≤60 years (3 of 32)	YES
Gorgoraptis et al. 2019	Prospective Cohort Study	Single moderate to severe	Mayo Classification	Road traffic accident n=18<br>Fall n=1<br>Assault n=2	TBI n=21 (14)<br>Controls n=11 (6)	TBI= 49 (median), 29-72 (range)<br>Controls= 57 (median), 29-72 (range)	TBI: (1) a history of a single moderate-severe TBI, (2) age over 18 years, (3) capacity to provide written informed consent, (4) no prior neurological or psychiatric illness, (5) no contraindication to PET or prior radiation exposure that when combined with the dose from the present study would exceed 10 mSv in addition to the natural background radiation in the previous 3 years, (6) no contraindication to MRI, and (7) no medication use or allergies that may compromise participant safety or interfere with study procedures<br>Controls: healthy subjects of roughly the same socioeconomic status and age	NS	18-51 years	PET imaging using flortaucipir (a.k.a. [18F]AV-1451 and [18F]T807) and total tau and phospho tau (p-Tau181) in CSF and blood using Quarterix	Flortaucipir binding was increased in right lateral occipital cortex in TBI v healthy controls and correlated with increases in total tau in cerebral cortical gray matter (ρ=0.53) but not in the white matter (ρ=0.44) and phospho-tau in cerebral white matter (ρ=0.52) but not in the gray matter (ρ =0.32) in CSF; No correlation found in healthy controls; Plasma T-tau concentration did not correlate with flortaucipir binding in either TBI or healthy control. There were no differences in T-tau or P-tau concentration in CSF or plasma between TBI and healthy controls	YES

NS= not stated, SD= standard deviation
