# Supplementary material for: Association between single moderate to severe traumatic brain injury and long-term tauopathy in humans and preclinical animal models: a systematic narrative review of the literature
Source: Acta Neuropathol Commun. 2022 Jan 31;10:13. doi: 10.1186/s40478-022-01311-0 (PMC8805270; doi:10.1186/s40478-022-01311-0)
Supplement: Supplementary file 4 — Additional file 4: This table discloses of the study characteristics for human based articles, including article title, study design, injury severity, injury rating, injury type, sample size, age at time of study for TBI and control populations, inclusion and exclusion criteria, post-TBI interval (time since injury), type of tau assessment, findings, and if those findings supported chronic tau development. [file 40478_2022_1311_MOESM4_ESM.pdf]

Table 1 cont. Human Study Characteristics.

Article	Study Design	Injury Severity	Injury Rating	Injury Type	Sample Size (n_{males})	Age (years)	Inclusion Criteria	Exclusion Criteria	Post-TBI Interval	Type of Tau Assessment	Findings	YES or NO Chronic Tau
Williams et al. 2018	Cross-sectional Study	Single moderate to severe	Ohio State University TBI Identification Method to assess total numbers of TBI, age of TBI, cause of TBI, and symptoms such as memory loss and LOC (moderate= LOC ≤ 30; severe= LOC ≤ 24hrs)	War-related	TBI n=8 (NS)** Controls n=18 (13)	TBI= 76.9 (mean), 11.9 (SD) Controls= 77.2 (mean), 8.2 (SD)	NS	Individuals with a previous penetrating head injury and those with medical conditions or hearing or vision loss severe enough to impede participation; those with low cognition MMSE <20	~29.4 years or ~43 years	ELISA on oligomeric tau (F9T and D11C) from sera	Single moderate to severe TBI group had significantly more oligomeric tau vs. controls	YES
Arena et al. 2020	Case-control Study	Single moderate to severe	NS	Assault n=1 Motor vehicle accident n=1 Fall n=1	TBI n= 3 (3) Controls n= 32 (25) (age-related tauopathies and primary tauopathies with no history of TBI)	TBI= 50s-70s (range) Controls= 50s-80s (range)	TBI: neuropathologically confirmed CTE Control: negative for history of TBI or participation in contact sports, neuropathological diagnosis of AD, Pick's Disease, or CBD; other controls were negative for history of TBI and non-demented with known age-related tau pathologies (PART, ARTAG and PART, or ARTAG	NS	Long-term (did not define this)	IHC using PHF-1, CP13, AT100, pS262, 3R, 4R, Tau-C3 (truncated tau at Aps421), and GT-7 and GT-38 (conformational tau)	long-term development of tau pathology is observed in long-term survivors of moderate to severe TBI, but does not appear to be different compared to age-related and primary tauopathies or AD just the pattern and distribution throughout the brain is distinct	YES
Okamura et al. 2019	Case Study	Single severe	NS	Prefrontal leucotomy of two schizophrenia patients	Schizophrenia leucotomized n=2 (1)	Case 1= 76 Case 2= 73	NS	NS	>53 years	IHC using AT8, AP422, RD3 and RD4, Gallyas Silver Stain, and immunoblot using pS396	Prefrontal leucotomy (model of severe TBI) leads to the development of long-term tau pathology around blood vessels and in neurons and astrocytes (CTE-like pathology) in the injured cortex but weaker in connecting regions; accumulation of both 3R and 4R tau was observed	YES
Tribett et al. 2019	Case Report	Single severe	NS	Shotgun injury to the brain	TBI n=1 male	63	NA	NA	42 years	IHC using AT8	AT8 p-tau immunoreactivity observed in the medial temporal lobe (entorhinal and transentorhinal Braak stage II); Foci of AT8 reactivity in neurons, astrocytes, and cell processes around blood vessels in irregular foci at depths of cortical sulci (criteria for CTE) were present near damaged brain and away from structural damage	YES
Kenney et al. 2018	Case Report	Single moderate to severe	DoD-VA criteria and assessed medical chart review	Motor vehicle accident	TBI n=1 female	63	NA	NA	24 years	IHC using AT8	Abundant NFT and tau dot-like lesions were predominantly found in the superficial layers of cerebral cortex, but were found throughout the deep gray and brainstem nuclei and were especially prominent in the mammillary bodies; AT8 immunoreactivity was observed in thorn-shaped astrocytes in the subpial regions and occasionally around small blood vessels in a CTE-like pattern	YES

NS= not stated, NA= not applicable, **= includes some with >1 TBI
