# Supplementary material for: Association between single moderate to severe traumatic brain injury and long-term tauopathy in humans and preclinical animal models: a systematic narrative review of the literature
Source: Acta Neuropathol Commun. 2022 Jan 31;10:13. doi: 10.1186/s40478-022-01311-0 (PMC8805270; doi:10.1186/s40478-022-01311-0)
Supplement: Supplementary file 5 — Additional file 5: This table discloses of the study characteristics for preclinical animal based articles, including article title, animal model, injury severity, injury rating, injury model, injury parameters, sample size, age of injury, post-TBI interval (time since injury), type of tau assessment, findings, and if those findings supported chronic tau development. [file 40478_2022_1311_MOESM5_ESM.pdf]

Table 2. Preclinical Animal Study Characteristics.

Article	Animal Model	Injury Severity	Injury Rating	Injury Model	Injury Parameters	Sample Size	Age of Injury	Post-TBI Interval	Type of Tau Assessment	Findings	YES or NO Chronic Tau
Zanier et al. 2018	C57BL/6J mice	Single severe	NS	CCI	-anesthetized with isoflurane inhalation<br>-rectal temperature maintained at 37º<br>-craniectomy<br>-injury induced with 3mm impactor at 20º angle from vertical<br>-injury applied between bregma and lambda over left parietotemporal cortex<br>-impactor velocity 5m/s<br>-deformation depth 1mm	TBI_{3mo} n=8*<br>$Sham_{3mo}$ n=7**<br>TBI_{12mo} n=7*<br>$Sham_{12mo}$ n=7**	2 months	3 months or 12 months	IHC and WB using AT8 and anti-total tau	3 out of 7 (~40%) TBI mice at 3mo developed abnormally phosphorylated tau ipsilateral to injury and only 1 out of 4 TBI mice showed increased total tau, while 6 out of 8 (~85%) TBI mice at 12mo developed abnormally phosphorylated tau both ipsilateral and contralateral to injury and 2 out of 3 TBI mice showed increased total tau levels	YES
Kondo et al. 2015	C57BL/6J mice	Single severe	NS	Weight Drop	-anesthetized with isoflurane inhalation for 45sec<br>-head placed under hallow guide tube<br>-impactor = 54g metal bolt<br>-drop height of 60in over dorsal aspect of skull<br>-rotational head acceleration through Kimwipe<br>-mice recovered in room air	TBI n=4<br>Sham n=4	2-3 months	6 months	Immunostaining using cis p-tau mAb, AT8, AT100, PHF1, and Alz50; WB using tau5 and cis p-tau	single severe TBI in WT mice given an IgG control led to chronic 6mo development of misfolded conformational tau (Alz50), hyperphosphorylated p-tau (AT100, PHF1, and AT8), pathological cis p-tau, increased total tau, and insoluble tau in hippocampus and cortex compared to TBI mice that received the *cis* mAb and sham mice	YES
Albayram et al. 2017	C57BL/6 mice	Single severe	NS	Weight Drop	-anesthetized with isoflurane inhalation for 45sec<br>-head placed under hallow guide tube<br>-impactor = 54g metal bolt<br>-drop height of 60in over dorsal aspect of skull<br>-rotational head acceleration through Kimwipe<br>-mice recovered in room air	TBI n=4-5<br>Sham n=4-5	2-3 months	6 months	Immunofluorescence against cis p-tau, T22 (oligomeric tau), AT8, and AT100	TBI mice that received IgG control at 6mo post-injury produced more cis p-tau, and T22 compared to sham mice and TBI mice that received *cis* mAb in mPFC and hippocampus and had more AT8 and AT100 positive staining compared to controls	YES
Tan et al. 2020	C56BL/6/SV129 WT mice	Single moderate	Apnoea, unconsciousness, and self righting reflex were timed from time of injury	FPI	-anesthetized with isoflurane inhalation<br>-3mm craniotomy over lateral parietal cortex<br>-hallow injury cap placed over craniotomy<br>-injury device attached to head cap on mouse<br>-FPI at force of 1-1.5atm	TBI n=4<br>Sham n=4	12 weeks	12 weeks	WB using p-tau (pS198 and pS396) and total tau (tau5)	TBI mice had significantly more pS198/Tau5 ratio compared to sham mice in the ipsilateral cortex but not with pS396/Tau5; no differences between the groups on total tau levels	YES
Glushakova et al. 2018	Sprague-Dawley rats	Single moderate to severe***	NS	CCI	-anesthetized with isoflurane inhalation<br>-rats placed in stereotactic apparatus with temperature-controlled heating pad<br>-5mm ipsilateral craniotomy between bregma and lambda in right parietal region<br>-4mm diameter impactor<br>-impact velocity 3.5m/s<br>-compression distance 2.5mm<br>-compression time 200ms<br>-rats recovered in temperature-controlled recovery chamber	TBI n=3-7<br>Sham n=3-7	230-300 gram rats (~2-3 months)	3 months	IHC using anti-caspase-3 cleaved tau (truncated at Asp421) antibody	Prominent perivascular accumulation of caspase-3 cleaved tau in corpus callosum 3mo post-injury and in both extracellular and cellular accumulations around cell nuclei or dead cells; Significantly more extracellular aggregates and perivascular aggregates in TBI compared to controls	YES

NS= not stated, CCI= controlled cortical impact, FPI= fluid percussion injury *= 3-4 mice were used for biochemical analysis and histology, **= n=2-5 mice were used for biochemical analysis and histology, ***= based on Siebold et al. 2018
