# Supplementary material for: Association between single moderate to severe traumatic brain injury and long-term tauopathy in humans and preclinical animal models: a systematic narrative review of the literature
Source: Acta Neuropathol Commun. 2022 Jan 31;10:13. doi: 10.1186/s40478-022-01311-0 (PMC8805270; doi:10.1186/s40478-022-01311-0)
Supplement: Supplementary file 6 — Additional file 6: This table discloses of the study characteristics for preclinical animal basedarticles, including article title, animal model, injury severity, injury rating, injury model, injuryparameters, sample size, age of injury, post-TBI interval (time since injury), type of tauassessment, findings, and if those findings supported chronic tau development. [file 40478_2022_1311_MOESM6_ESM.pdf]

Table 2. cont. Preclinical Animal Study Characteristics.

Article	Animal Model	Injury Severity	Injury Rating	Injury Model	Injury Parameters	Sample Size	Age of Injury	Post-TBI Interval	Type of Tau Assessment	Findings	YES or NO Chronic Tau
Shultz et al. 2015	Long-Evans rats	Single severe	Apnoea, unconsciousness, and self righting reflex were timed from time of injury	Lateral FPI	-anesthetized with isoflurane inhalation for 3min<br>-5mm diameter craniotomy centered -3mm posterior and 4mm lateral of bregma<br>-hallow injury cap placed over craniotomy<br>-injury device attached to head cap on rat<br>-FPI pulse of 3atm	$TBI_{saline-vehicle}$ n=13<br>$Sham_{saline-vehicle}$ n=12	12 weeks	12 weeks	WB using anti-pS198, anti-pS262, and anti-tau5	Rats given FPI+saline vehicle treatment had significantly increased ratio of pS198/total tau in the injured cortex compared to sham-injured but not the ratio of pS262/total tau and the amount of total tau in FPI groups was significantly decreased compared to sham-injured regardless of treatment; expression levels of pS198 and pS262 was not significantly different	*YES*
Acosta et al. 2017	Sprague-Dawley rats	Single moderate	NS	CCI	-anesthetized with isoflurane inhalation<br>-2.5mm radius craniectomy from bregma at -0.2 anterior and +0.2mm lateral right (fronto-parietal cortex)<br>-impactor rod placed 15º vertically<br>-impact velocity 6m/s<br>-impact depth 1mm<br>-dwell time 150ms<br>-thermal blanket pad and rectal thermometer to maintain body temperature	TBI n=14<br>Sham n=6	2 months	6 months	Immunofluorescence using AT8 and T22 (oligomeric tau)	significant accumulation of p-tau AT8 in ipsilateral cortex and dentate gyrus vs. sham and significant accumulation of oligomeric tau T22 in ipsilateral and contralateral cortex and dentate gyrus vs. sham; pathology in the ipsilateral cortex and dentate gyrus of TBI mice was significantly higher than the contralateral hemispheres	YES
Edwards et al. 2020	P301S mice	Single moderate to severe	Latency of foot, tail, and righting reflexes	CCI	-anesthetized with isoflurane inhalation<br>-5mm diameter craniotomy on right parietal cortex between bregma and lambda<br>-impact velocity 3m/s<br>-impact deformation 1mm<br>-mice recovered in warm chamber	TBI n=4-6<br>Sham n=4-6	3 months	6 months	IHC using AT8	AT8 p-tau increased in amount and extent compared to sham in the ipsilateral and contralateral cortex and hippocampus	YES
Kokiko-Cochran et al. 2018	hTau and C57BL/6J	Single moderate	NS	Lateral FPI	-anesthetized with ketamine and xylazine<br>-3mm craniotomy over right parietal cortex midway between bregma and lambda<br>-Leur lock syringe placed over exposed dura<br>-animals allowed to recover on heating pad<br>-24hrs later, mice anesthetized with ketamine and xylazine and connected to FPI device<br>-FPI at average force of 1atm<br>-animals recovered on heating pad	TBI n=5-6<br>Sham n=5-6	2 months	135 days (~4 months)	IHC using AT180 and Gallyas silver stain	TBI significantly increased AT180 immunoreactivity in hTau TBI mice in the lateral cortex compared to other groups; in the ipsilateral temporal cortex, hTau TBI mice had significantly more AT8 immunoreactivity (mean= 6.14) compared to B6 TBI (mean= 1.81) and sham but not compared to hTau sham mice (mean= 1.93); significantly more Gallyas+ cells in the ipsilateral hippocampus of hTau TBI mice compared to B6 TBI mice	YES

NS= not stated, CCI= controlled cortical impact, FPI= fluid percussion injury, *italicized indicate interpret with caution*
